# Tris(2-Pyridyl)Arsine as a New Platform for Design of Luminescent Cu(I) and Ag(I) Complexes

**DOI:** 10.3390/molecules27186059

**Published:** 2022-09-16

**Authors:** Yan V. Demyanov, Evgeniy H. Sadykov, Marianna I. Rakhmanova, Alexander S. Novikov, Irina Yu. Bagryanskaya, Alexander V. Artem’ev

**Affiliations:** 1 Nikolaev Institute of Inorganic Chemistry, SB RAS, 3 Acad. Lavrentiev Ave., 630090 Novosibirsk, Russia; 2 Saint Petersburg State University, Universitetskaya Nab. 7/9, 199034 Saint Petersburg, Russia; 3 Peoples’ Friendship University of Russia (RUDN University), Miklukho-Maklaya Street 6, 117198 Moscow, Russia; 4 N. N. Vorozhtsov Novosibirsk Institute of Organic Chemistry, SB RAS, 9 Acad. Lavrentiev Ave., 630090 Novosibirsk, Russia

**Keywords:** tris(2-pyridyl)arsine, Cu(I) complexes, Ag(I) clusters, metallophilic interactions, thermally activated delayed fluorescence, dimerization

## Abstract

The coordination behavior of tris(2-pyridyl)arsine (Py_3_As) has been studied for the first time on the example of the reactions with CuI, CuBr and AgClO_4_. When treated with CuI in CH_2_Cl_2_ medium, Py_3_As unexpectedly affords the scorpionate complex [Cu(Py_3_As)I]∙CH_2_Cl_2_ only, while this reaction in MeCN selectively leads to the dimer [Cu_2_(Py_3_As)_2_I_2_]. At the same time, the interaction of CuBr with Py_3_As exclusively gives the dimer [Cu_2_(Py_3_As)_2_Br_2_]. It is interesting to note that the scorpionate [Cu(Py_3_As)I]∙CH_2_Cl_2_, upon fuming with a MeCN vapor (r.t., 1 h), undergoes quantitative dimerization into the dimer [Cu_2_(Py_3_As)_2_I_2_]. The reaction of Py_3_As with AgClO_4_ produces complex [Ag@Ag_4_(Py_3_As)_4_](CIO_4_)_5_ featuring a Ag-centered Ag_4_ tetrahedral kernel. At ambient temperature, the obtained Cu(I) complexes exhibit an unusually short-lived photoluminescence, which can be tentatively assigned to the thermally activated delayed fluorescence of (M + X) LCT type (M = Cu, L = Py_3_As; X = halogen). For the title Ag(I) complexes, QTAIM calculations reveal the pronounced argentophilic interactions for all short Ag∙∙∙Ag contacts (3.209–3.313 Å).

## 1. Introduction

Over the past decade, luminescent Cu(I) and Ag(I) complexes have attracted a considerable attention due to their intriguing structural diversity [1,2,3,4,5,6,7,8,9,10,11] and ability to exhibit efficient phosphorescence or thermally activated delayed fluorescence (TADF) [12,13,14,15,16,17,18,19], or even dual emission [20,21]. Owing to these features, such compounds are now considered as promising emitters for energy-efficient OLEDs of second and third generations (PHOLED and TADF OLED) [22,23], just as for light-emitting electrochemical cells (LEECs) [24]. Moreover, Cu(I) and Ag(I) complexes are reported to be perspective luminescent sensors and X-ray scintillators, as well as “smart” materials, the emission of which is sensitive to the external stimuli (temperature, pressure, chemicals) [25,26,27,28].

To design luminescent Cu(I) and Ag(I) complexes, various C-, N-, and P-donor ligands such as carbenes, azaheterocycles, and phosphines are commonly exploited [29,30,31,32,33,34]. At that, “heavy pnictine(III)”-based ligands, e.g., arsines, remain almost unexplored in this regard. Meanwhile, recent studies [35,36,37] have demonstrated that the arsine ligands can be preferable over similar phosphines to fabricate Cu(I)-based TADF materials [38]. Indeed, the higher spin-orbital coupling (SOC) strength of arsenic (ζ_l_ = 1202 cm^−1^) [39] against phosphorus (ζ_l_ = 230 cm^−1^) [39] makes the emission rate of Cu(I)-arsine complexes much faster compared to that of similar Cu(I)-phosphine derivatives [38]. Considering that the triplet or TADF emitters with short decay times (<2 μs) are essential for OLED related applications, the design of new Cu(I)-arsine complexes, just as their Ag(I) congeners, represents a daunting challenge.

Working in this field, we have paid attention to pyridylarsines, “heavy pnictogen” analogs of pyridylphosphines. The latter are a famous family of ligands which are widely used for the design of Cu(I) and Ag(I) complexes showing very efficient TADF or/and phosphorescence [40,41,42,43,44]. The survey of the literature reveals, however, that the azaheterocyclic-substituted arsines are very limited in number [45,46,47], and their Cu(I)/Ag(I) derivatives are even scarcer [38,46,47]. Herein, for the first time, we have employed tris(2-pyridyl)arsine as a stabilizing platform for Cu(I) and Ag(I) complexes. The latter have been studied in terms of solid-state luminescence, thermal stability and electronic structure.

## 2. Results and Discussion

### 2.1. Synthesis and Characterization 

**Scheme 1:**
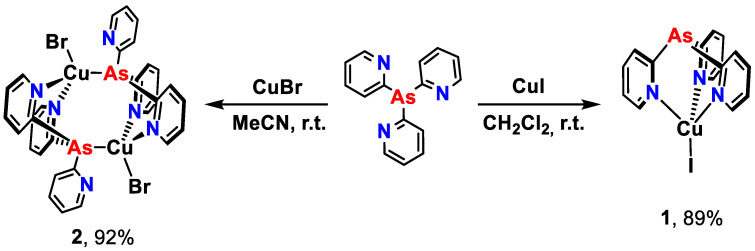
Reactions of Py_3_As with CuBr and CuI.

It was reported that tris(2-pyridyl)phosphine (Py_3_P) reacted with Cu(I) halides to irreversibly give air-stable dimeric complexes [Cu_2_(Py_3_P)_2_X_2_] (X = Cl, Br, I) in high yields [48]. Therefore, it would be reasonable to expect that Py_3_As could also give the related complexes. Meanwhile, we have found that the reactivity of Py_3_As differs from that of Py_3_P. Namely, CuI easily reacts with Py_3_As in CH_2_Cl_2_ to furnish the unexpected scorpionate [Cu(Py_3_As)I] (**1**) only, isolated as a solvate **1**·CH_2_Cl_2_ in 89% yield (Scheme 1). By contrast, CuBr under similar conditions affords a dimeric complex [Cu_2_(Py_3_As)_2_Br_2_] (**2**) in 92% yield. Our numerous attempts to synthesize CuBr-based scorpionate under the varied conditions (different Cu/Py_3_As ratios, diverse solvents) failed: complex **2** was already formed in all the experiments. Of note, CuCl also easily reacts with Py_3_As, but a product formed was found to be easily oxidized in air to produce unidentified green Cu(II) complexes.

To explain the different reactivity of Py_3_As towards CuBr and CuI, the dimerization energies for the equilibriums 2[Cu(Py_3_As)X] ↔ [Cu_2_(Py_3_As)_2_X_2_] (X = Br, I) have been assessed at the PBE0/def2-TZVP level of the theory. The performed calculations reveal that the formation of a dimer is indeed thermodynamically more preferable for X = Br by 2.34 kcal∙mol^–1^, whereas scorpionate is favorable for X = I by 0.97 kcal∙mol^–1^ (Supplementary Materials, Figure S10). It should be noted, however, that an additional stabilization of scorpionate [Cu(Py_3_As)I] in **1**·CH_2_Cl_2_ is possible through the solvate molecules (*vide infra*).

**Figure 1:**
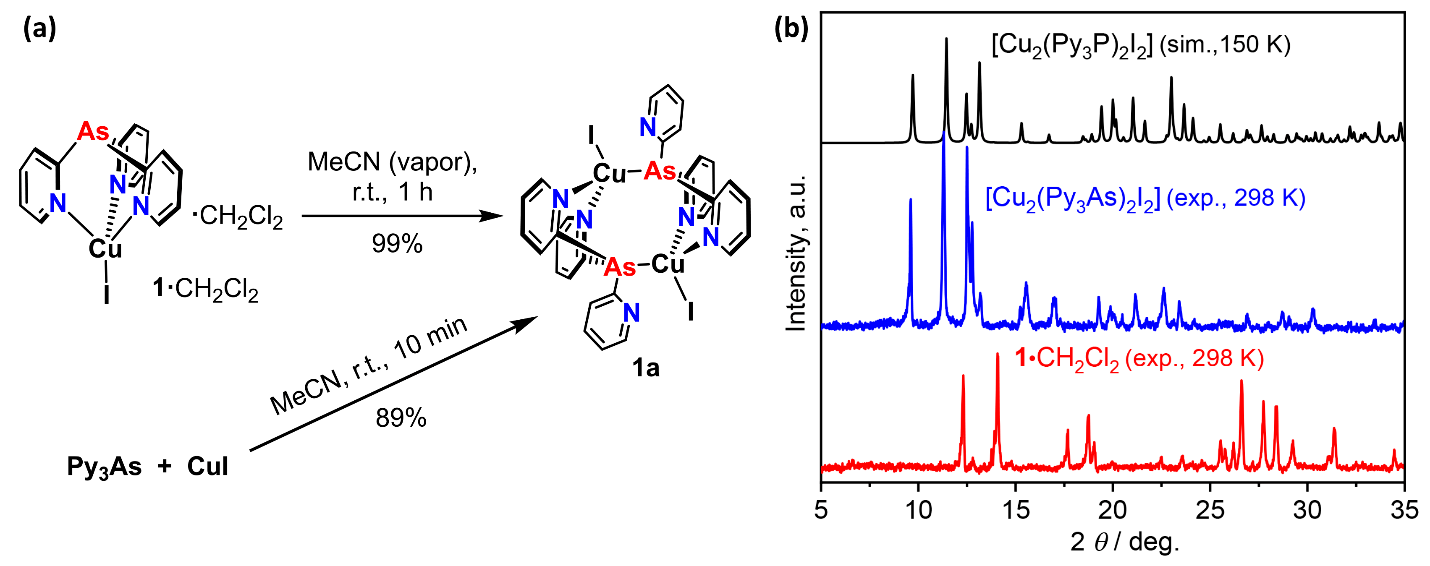
(**a**) MeCN-driven dimerization of **1**, and direct interaction of Py_3_As with CuI in MeCN; (**b**) associated changing in PXRD patterns: experimental diffractograms of starting complex **1** (down curve), product **1a** (middle curve), and simulated PXRD pattern for [Cu_2_(Py_3_P)_2_I_2_] (top curve).

Our attempts to transform **2** into scorpionate [Cu(Py_3_As)Br] using recrystallization from different solvents failed. Meanwhile, we have found that the fuming of **1**·CH_2_Cl_2_ with MeCN vapor (23 °C, 1 h) results in quantitative and irreversible dimerization into [Cu_2_(Py_3_As)_2_I_2_] (Figure 1a). The reaction is accompanied by the changing of the parent emission color of **1** (*vide infra*) to green, which is specific for complexes of [Cu_2_(Py_3_As)_2_X_2_] type (X = P or As). The powder X-ray diffraction (PXRD) pattern of the product [Cu_2_(Py_3_As)_2_I_2_], differs from that of [Cu_2_(Py_3_As)_2_Br_2_] (**2**), but closely resembles that of a similar phosphine derivative, [Cu_2_(Py_3_P)_2_Br_2_] (Figure 1b). Since the attempts to grow X-ray quality crystals of **1a** met with no success, its structure has been proved by PXRD, mid-IR and ^1^H NMR data only. Eventually, we have found that complex **1a** can be successfully synthesized in an almost quantitative yield by the straightforward reaction of Py_3_As with CuI in MeCN (r.t., 10 min) (Figure 1a). Thus, a noticeable effect of solvents on the reaction outcome has been established for the reaction of Py_3_As with CuI.

**Scheme 2:**
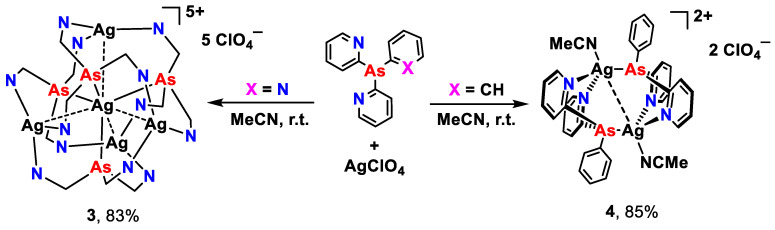
Reactivity of Py_3_As and Py_2_PhAs in reactions with AgClO_4_.

In the next step, we have examined the complexation of Py_3_As with Ag(I) using AgClO_4_ as a precursor. The reaction easily occurs in MeCN medium, and the following recrystallization of the product in water produces a fournuclear cluster [Ag@Ag_4_(Py_3_As)_4_](CIO_4_)_5_·3H_2_O (**3**·3H_2_O) in high yield (Scheme 2). Of note, the same product is also formed even when the AgClO_4_/Py_3_As molar ratio deviates from the stoichiometric one (5:4) being 1:1, 2:1, or 1:2. For comparison, the reaction of bis(2-pyridyl)phenylarsine (Py_2_PhAs) with AgClO_4_ under similar conditions has also been implemented to deliver a dinuclear complex [Ag_2_(Py_2_PhAs)_2_(MeCN)_2_](CIO_4_)_2_·CH_3_CN (**4**·CH_3_CN), the structure of which resembles that of **2**. Again, the AgClO_4_/Py_2_PhAs ratio does not affect the reaction outcome: only complex **2** is formed.

**Figure 2:**
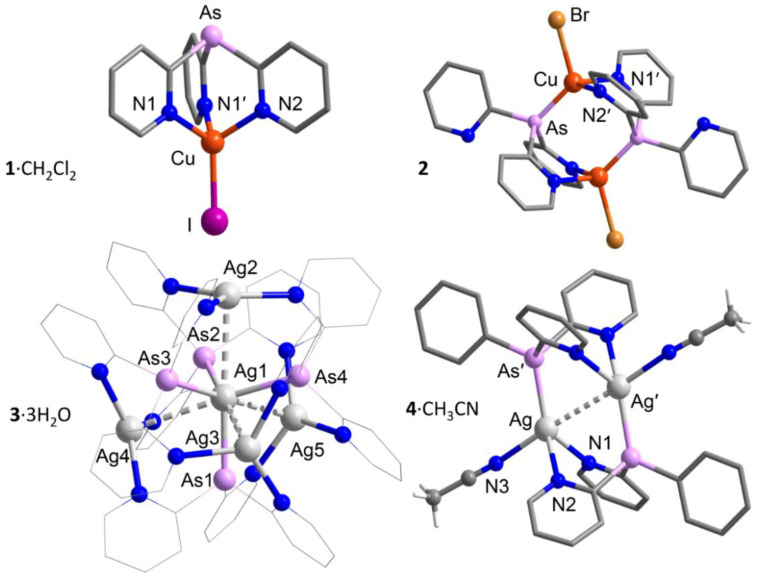
X-ray derived structures of **1**·CH_2_Cl_2_, **2**, **3**·3H_2_O (one of three independent parts) and **4**·CH_3_CN. The aromatic H atoms, solvate molecules and counterions are omitted for clarity.

The X-ray derived structures of the prepared complexes are displayed in Figure 2, and the selected interatomic distances are listed in Table 1. In the structure of **1**·CH_2_Cl_2_ (Figure 2), the Cu atom is coordinated by Py_3_As in a N,N′,N″-tripodal manner, and the iodine atom completes a distorted tetrahedral environment of the metal (τ_4_ = 0.85) [49]. Note that the iodine atom of **1** deviates from the axis passing from As and Cu atoms by only 0.8°, unlike the similar complex with tris(2-pyridyl)arsine oxide, where such deviation reaches 9.4° [50]. The dihedral angles between averaged pyridine planes of **1** are ≈ 114.3°, 114.4° and 131.2°.

Complex **2** consists of two CuBr units bridged by two Py_3_As ligands (μ_2_-As,N,N’) in a head-to-tail manner so that an inversion center is located in the middle of the molecule (Figure 2). Therefore, each Cu atom adopts a distorted tetrahedral [Cu@AsN_2_Br] environment (τ_4_ = 0.90). The intramolecular Cu∙∙∙Cu distances (*ca*. 3.94 Å) are too long for the appearance of metallophilic interactions (*cf.* twice van der Waals radius of Cu is 2.80 Å [51]). The dihedral angle between planes of the coordinated pyridine rings of **2** is about 124.8°. Overall, the structure of **2** closely resembles that of similar Py_3_P-based complex [Cu_2_(Py_3_P)_2_Br_2_] [48].

Crystals of **3**·3H_2_O contain three independent [Ag@Ag_4_(Py_3_As)_4_]^5+^ cations charge-balanced by non-coordinated ClO_4_^−^ anions, as well as the lattice water molecules. The independent [Ag@Ag_4_(Py_3_As)_4_]^5+^ cations have a similar structure, which consists of a *C_3_*-symmetrical Ag@Ag_4_ tetrahedral kernel supported by four Py_3_As ligands. The central Ag atom adopts a slightly distorted tetrahedral geometry (τ_4_ = 0.98) constituted of four As atoms. Each vertex Ag atom of the Ag@Ag_4_ kernel is coordinated by three N atoms of three neighboring Py_3_As ligands, thus accepting a trigonal pyramidal geometry [3 + 1]. Considering that the average distance between central and vertex Ag atoms of Ag@Ag_4_ kernel (3.25 Å) is shorter than twice van der Waals Ag radius (3.44 Å) [51], argentophilic interactions could occur (*vide infra*). To sum up, Py_3_As ligands in **3** exhibit an As,N,N′,N″-coordination manner that is similar to that of Py_3_P in [Ag@Ag_4_(Py_3_P)_4_]^5+^ complexes [52].

The structure of the cationic part of **4**·CH_3_CN, [Ag_2_(Py_2_PhAs)_2_(MeCN)_2_]^2+^, is formed by two Ag(I) cations bridged by two Py_2_AsPh ligands in a head-to-tail manner. Both metal cations are also ligated by MeCN, thereby adopting a distorted trigonal pyramidal geometry. Again, the Ag…Ag distance in **4** being 3.2206(4) Å implies argentophilic interaction, which is actually taking pace according to the theoretical calculations (*vide infra*).

The synthesized compounds are moderately soluble in dichloromethane and acetonitrile (**1**·CH_2_Cl_2_ and **2**), or in water (**3**·3H_2_O and **4**·CH_3_CN). All of them are air-stable, except for **4**·CH_3_CN, which quickly loses the coordinated acetonitrile molecules upon storage in air. The ^1^H NMR spectra of **1**–**4** demonstrate one set of signals from the coordinated arsines and ancillary ligands (Figures S3–S7), indicating the existence of symmetrical species in a solution (closely uninvestigated). The mid-IR spectra of these complexes show characteristic bands of the coordinated pyridyl-containing arsenic ligands (Figure S8). Moreover, complexes **3**·3H_2_O and **4**·CH_3_CN display a broad band at 1097–1099 cm^−1^ belonged of ν_Cl–O_ stretching vibrations of free ClO_4_^−^ anions, and specific bands from H_2_O and MeCN molecules/ligands, respectively, i.e., ν_O–H_ = 3610 cm^−1^ and ν_C≡N_ = 2268 cm^−1^.

The thermal stability of the above complexes has been studied by TGA, DTG and DTA techniques under argon atmosphere (Figure S9). The solvate molecules of **1**·CH_2_Cl_2_ are lost at the range of 100–127 °C, but complex **1** itself is stable up to ≈200 °C. A higher stability is inherent in **2**, which begins to decompose at about 240 °C. Compounds **3**·3H_2_O and **4**·CH_3_CN lose the solvate molecules at 120–140 °C and 135–155 °C, respectively, after which they remain stable at least up to 270 °C.

### 2.2. Theoretical Consideration

**Figure 3:**
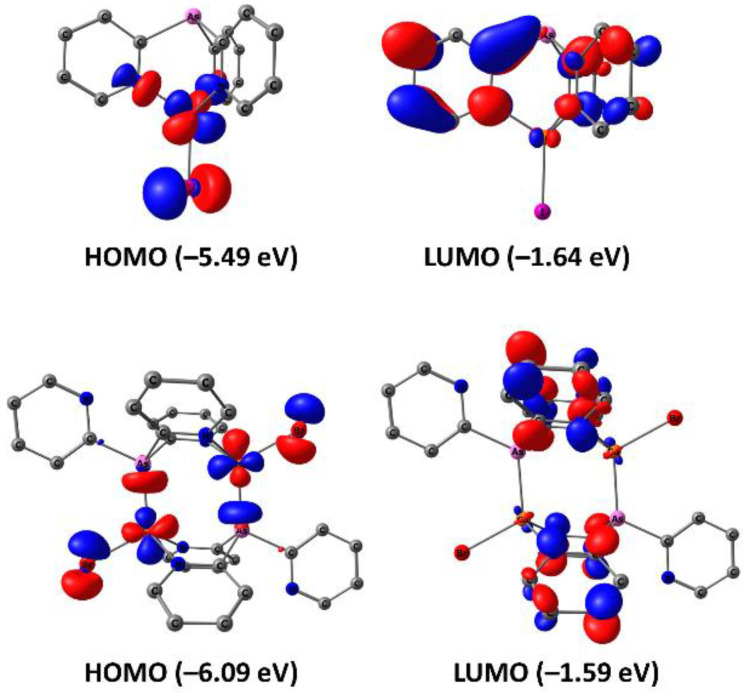
Frontier orbitals of **1** and **2** calculated at PBE0/def2TZVP level (isosurface = 0.045).

The electronic structure of luminescent Cu(I) complexes **1** and **2** has been investigated at the PBE0/def2TZVP level of the theory (for details, see §6 in ESI) to understand electronic transitions responsible for the excitation. For both complexes, the highest occupied molecular orbital (HOMO) and nearby HOMO-n are largely contributed by metal’ d-orbitals and the lone pairs in halogen atoms (Figure 3, Figures S11, S12; Tables S2, S3). The lowest unoccupied molecular orbital (LUMO) and nearby LUMO+n are mainly π-orbitals on the pyridine rings (Figure 3, Figures S11, S12; Tables S2, S3). Interestingly, a lone pair in As atom does not contribute to the highest MOs (HOMO–HOMO-6). The fact that the frontier MOs of **1** and **2** are well separated in the space indicates a quite small energy gap between the lowest singlet (S_1_) and triplet (T_1_) exited states. Overall, the predicted HOMO/LUMO distribution is very typical for emitting TADF Cu(I) halide complexes. 

A detailed consideration of HOMOs of **1** and **2** reveals small energy gaps separation between the HOMO and HOMO-1 levels which are, moreover, populated by d-orbitals with different spatial orientations (Figures S11 and S12). In particular, the HOMO-1/HOMO separation is 140 cm^−1^ for **2**, and it is just 2 cm^−1^ for **1**. According to the literature [12,13], such a scenario demonstrates a strong SOC mixing of the S_1_ and T_1_ states, which are originated from HOMO-1 → LUMO and HOMO → LUMO transitions, respectively. This, in turn, accelerates the rates of both S_1_ → T_1_ and T_1_ → S_1_ spin-forbidden processes, and hence, increases total rate of the luminescence. The experimental results fully confirm these predictions (*vide infra*). TD-DFT computations of **1** and **2** testify to the (M + X) LCT character (X = Br or I) of the low-energy absorptions (Tables S4, S5) that is specific for the related complexes. Furthermore, the (M + X) LCT nature of the lowest excited states is confirmed by a specific spin distribution in the computed T_1_ state of **1** (Figure S15). The lowest (LSOMO) and highest (HSOMO) single occupied molecular orbitals of the T_1_ state of **1** (Figure S15) closely resemble HOMO and LUMO in its S_0_ state (Figure 3). The calculated ∆E(T_1_–S_0_) energy gap for the optimized states of **1** being 1.81 eV reasonably agrees with the emission energy of **1**·CH_2_Cl_2_ at a pure phosphorescence regime (77 K), i.e., 1.98 eV or 625 nm (*vide infra*). Therefore, one can expect that the emitting excited states of the compounds discussed should be of the (M + X) LCT type.

**Figure 4:**
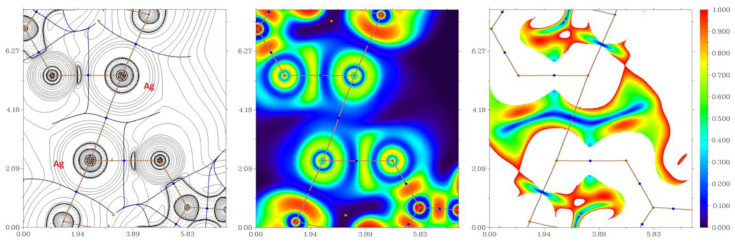
Contour line diagram of the Laplacian of electron density distribution ∇^2^ρ(**r**), bond paths, and selected zero-flux surfaces (left panel), visualization of electron localization function (ELF, center panel) and reduced density gradient (RDG, right panel) analyses for metallophilic interactions in complex **4**. Bond critical points (3, –1) are shown in blue, nuclear critical points (3, –3)—in pale brown, ring critical points (3, +1)—in orange, bond paths are shown as pale brown lines, length units – Å, and the color scale for the ELF and RDG maps is presented in a.u.

For Ag(I) complexes **3** and **4**, which appear to be almost non-emissive, argentophilic interactions have been examined by QTAIM (quantum theory “atoms in molecule”) method at the ωB97XD/DZP-DKH level (for details, see §6.3 in ESI). The QTAIM analysis of model structures reveals the presence of the bond critical points (3, –1) for metallophilic interactions in **3** and **4** (Table 2). The low magnitude of the electron density (0.017–0.021 a.u.), positive values of the Laplacian of electron density (0.028–0.030 a.u.), and negative energy density (from –0.002 to –0.003 a.u.) in the bond critical points (3, –1) for Ag(I)···Ag(I) interactions in **3** and **4** are typical for metallophilic interactions in other metal complexes [53,54,55,56,57,58,59]. The balance between the Lagrangian kinetic energy G(**r**) and potential energy density V(**r**) at the bond critical points (3, –1) for Ag(I)···Ag(I) interactions in **3** and **4** [*viz.* –G(**r**)/V(**r**) < 1] shows some covalent contribution in these short contacts [60]. The Laplacian of electron density is typically decomposed into the sum of contributions along the three principal axes of maximal variation, giving three eigenvalues of the Hessian matrix (λ_1_, λ_2_ and λ_3_), and the sign of λ_2_ can be utilized to distinguish the bonding (attractive, λ_2_ < 0) weak interactions from the non-bonding ones (repulsive, λ_2_ > 0) [61,62]. Thus, the Ag(I)···Ag(I) interactions in **3** and **4** have an attractive character. For illustration, the calculated electron density distribution at the metal atoms of **4** is plotted in Figure 4.

### 2.3. Photoluminescence of 1 and 2

**Figure 5:**
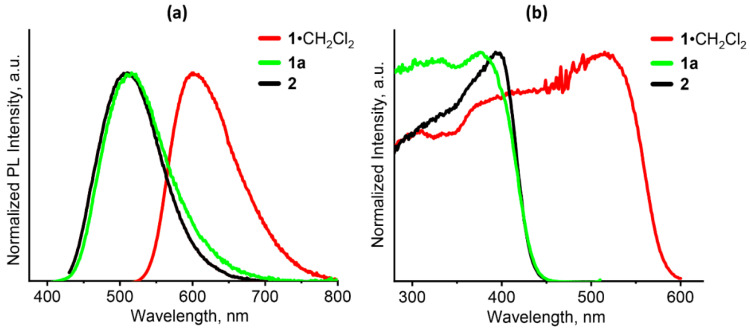
Emission (**a**) and excitation (**b**) spectra of **1**·CH_2_Cl_2_, **2** and **3**·3H_2_O at 300 K. The emission spectra were recorded at λ_ex_ = 390 nm (for **1a**, **2**) and 500 nm (**1**·CH_2_Cl_2_).

At ambient temperature, Cu(I) complexes **1**, **1a** and **2** emit pronounced solid state photoluminescence (PL), whereas Ag(I) derivatives **3** and **4** appear to be almost non-emissive. The recorded emission and excitation spectra of **1**, **1a** and **2** are shown in Figure 5, and the measured PL properties are given in Table 3. As follows from these data, scorpionate **1** manifests an orange PL, while dimers **1a** and **2** emit in a green region. In the terms of PL quantum yields (PLQYs), the emission of the studied compounds is moderate at 298 K (10–14%). All the emission profiles are of broad and structureless shape that is inherent in PL of the charge transfer origin [14]. The corresponding excitation curves are displayed by typical bands extending from the UV-edge and are sharply falling close at ≈450 nm (**1a**, **2**) or 590 nm (**1**). The fact that the emission and emission profiles of iodide **1a** and bromide **2** are almost superimposable is not confusing; previously, the similar cases were documented for other halide complexes, including the related ones [38,48,63]. The PL lifetimes of **1**, **1a** and **2** at 298 K are remarkably short (0.8–1.9 μs) compared to the most known Cu(I) complexes. Accordingly, the radiative constants (k_r_ = PLQY/τ) being (0.53–1.75)∙10^5^ s^–1^ are relatively high, thereby indicating a strong SOC effect that is also predicted by DFT calculations (*vide supra*). For comparison, radiative rates (k_r_) of the similar Py_3_P-based complexes [Cu_2_(Py_3_P)_2_X_2_] at 298 K are much lower: 2.9∙10^4^ and 2.6∙10^4^ s^–1^ for X = Br and I, respectively. The acceleration of the emission rates observed in the arsine complexes is obviously attributed to much stronger SOC effect of arsenic compared to that of phosphorus. Previously, this effect was already demonstrated for Cu(I)-arsine complexes [38].

**Figure 6:**
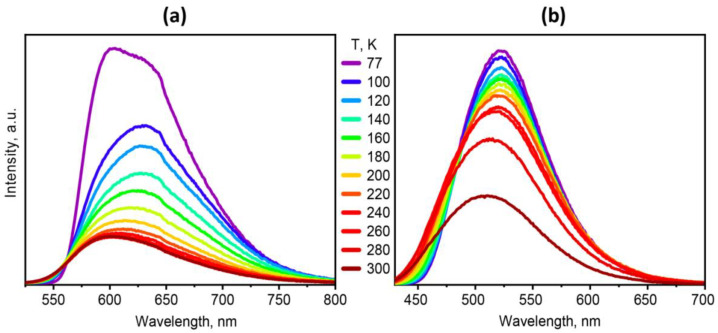
Temperature dependent emission spectra of **1**·CH_2_Cl_2_ (**a**) and **2** (**b**) recorded at λ_ex_ = 500 and 380 nm, respectively.

Temperature-dependent PL spectra of **1** and **2** (Figure 6) demonstrate the significant enhancement of PL intensity upon cooling that is accompanied by a slight bathochromic shift of the bands by 10–20 nm (Table 3). When passing from 298 to 77 K, the PL lifetimes of **1** and **2** increase by 13 and 25 times, thus amounting 25 and 23 μs at 77 K, respectively. Taken together, these observations suggest the TADF manifestation at ambient temperature and phosphorescence at 77 K. According to the DFT and TD-DFT computations, the ^1^(M + X) LCT emissive state is responsible for TADF (298 K), and ^3^(M + X) LCT state is active at the phosphorescence regime (77 K). It should be underlined that the same emission scheme was previously proved for the related dimeric complexes based on Py_3_P and PhPy_2_As ligands. The ∆E_ST_ energy gaps between the ^1^(M + X) LCT and ^3^(M + X) LCT states of **1** and **2** can be roughly estimated by the red-shifting of the left flank of emission bands at their half height (Figure S17). The estimated ∆E_ST_ gaps, being 340 and 890 cm^–1^ for **1** and **2**, respectively, fall close to the range of such values (∆E_ST_ < 1500 cm^–1^) for TADF-active Cu(I) complexes [12,13,14].

## 3. Materials and Methods

### 3.1. General

All synthetic procedures were carried out under an argon atmosphere using the standard Schlenk technique. CuI (≥99%, Sigma, Gillingham, UK), AgClO_4_ (97%, Alfa Aesar, Heysham, UK), and MeCN (HPLC grade, Cryochrom, St. Petersburg, Russia) were used as purchased. Prior to use, CuBr was freshly synthesized by the treatment of CuBr_2_ with Cu powder in MeCN solution. Tris(2-pyridyl)arsine, [50] and bis(2-pyridyl)phenylarsine (Py_2_AsPh) [38] were prepared according to the literature procedures. ^1^H NMR spectra were recorded using a Bruker AV-500 spectrometer at 500.13 MHz. Chemical shifts were reported in δ (ppm) relative to residual peaks of protonated CDCl_3_, DMSO-d^6^, and CD_3_CN. FT-IR spectra were recorded on a Bruker Vertex 80 spectrometer. Powder X-ray diffraction patterns were recorded on a Shimadzu XRD-7000 diffractometer (Cu-Kα radiation, Ni – filter, 3–35° 2*θ* range, 0.03° 2*θ* step, 5s per point). Thermogravimetric analyses (TGA – c-DTA – DTG) were carried out in a closed Al_2_O_3_ pan under argon flow at a 10 °C/min^–1^ heating rate using a NETZSCH STA 449 F1 Jupiter STA. CHN microanalyses were performed on a MICRO cube analyzer.

Emission and excitation spectra were recorded on a Fluorolog 3 spectrometer (Horiba Jobin Yvon) equipped with a cooled PC177CE-010 photon detection module and an R2658 photomultiplier. The absolute PLQYs were determined at 298 K using a Fluorolog 3 Quanta-phi integrating sphere. Temperature-dependent excitation and emission spectra as well as emission decays were recorded using an Optistat DN optical cryostat (Oxford Instruments) integrated with the above spectrometer.

### 3.2. [Cu(Py_3_As)I]·CH_2_Cl_2_ (**1**·CH_2_Cl_2_)

A mixture of CuI (16.5 mg, 0.087 mmol) and Py_3_As (30 mg, 0.097 mmol) in CH_2_Cl_2_ (2 mL) was stirred at room temperature for 10 min. To the resulting solution, hexane (1 mL) was added dropwise and a precipitate formed was centrifuged and dried in air. Orange powder. Yield: 39 mg (89%). Single crystals of **1**·CH_2_Cl_2_ were grown by slow evaporation of CH_2_Cl_2_ solution for overnight. ^1^H NMR (500.13 MHz, CDCl_3_, ppm), δ: 9.09 (ddd, *J* = 5.0 Hz, *J* = 1.9 Hz, *J* = 0.9 Hz, 3H, H^6^ in Py), 7.90 (dt, *J* = 7.5 Hz, *J* = 1.2 Hz, 3H, H^4^ in Py), 7.71 (dt, *J* = 7.6 Hz, *J* = 1.8 Hz, 3H, H^5^ in Py), 7.35 (ddd, *J* = 7.7 Hz, *J* = 5.0 Hz, *J* = 1.3 Hz, 3H, H^3^ in Py), 5.31 (s, 2H in CH_2_Cl_2_). FT-IR (KBr, cm^–1^): 409 (m), 457 (w), 482 (s), 617 (w), 638 (w), 669 (w), 702 (m), 733 (s), 758 (vs), 773 (m), 787 (m), 897 (vw), 1003 (m), 1045 (m), 1088 (w), 1103 (w), 1152 (m), 1227 (w), 1271 (m), 1422 (s), 1447 (vs), 1553 (m), 1572 (s), 1636 (vw), 2955 (w), 3028 (w), 3042 (w). Calculated for C_16_H_14_AsCuICl_2_N_3_ (584.58): C, 32.9; H, 2.4; N, 7.2. Found: C, 33.0; H, 2.5; N, 7.2.

### 3.3. [Cu_2_(Py_3_As)_2_I_2_] (**1a**)

*Method 1:* A solid sample of **1**·CH_2_Cl_2_ (15 mg, 0.026 mmol) was placed in a 3 mL vial, which was then placed in a closed 50 mL weighing bottle containing MeCN (≈0.5 mL) on a bottom. Exposure of a solid sample **1**·CH_2_Cl_2_ under MeCN vapor at ambient temperature for 1 h results in the formation of **1a** as an off-white solid. Yield: 99% (12.5 mg).

*Method 2:* A mixture of CuI (8.5 mg, 0.045 mmol) and Py_3_As (15 mg, 0.049 mmol) in CH_3_CN (1 mL) was stirred at room temperature for 10 min. The formed precipitate was centrifuged and dried in vacuum. White powder. Yield: 40 mg (89%).

^1^H NMR (500.13 MHz, CD_3_CN, ppm), δ: 8.88 (d, *J* = 5.0 Hz, 6H, H^6^ in Py), 8.03 (d, *J* = 7.6 Hz, 6H, H^4^ in Py), 7.85 (t, *J* = 7.7 Hz, 6H, H^5^ in Py), 7.49-7.43 (m, 6H, H^3^ in Py). FT-IR (KBr, cm^–1^): 405 (w), 419 (m), 459 (w), 474 (m), 484 (s), 617 (w), 637 (m), 741 (w), 756 (vs), 779 (m), 988 (m), 1007 (m), 1049 (m), 1088 (w), 1103 (w), 1119 (w), 1155 (m), 1227 (w), 1275 (m), 1410 (s), 1423 (vs), 1447 (vs), 1558 (s), 1570 (s), 1578 (s), 1634 (w), 2974 (m), 3038 (m), 3059 (m). Calculated for C_30_H_24_As_2_Cu_2_I_2_N_6_ (999.29): C, 36.1; H, 2.4; N, 8.4. Found: C, 36.0; H, 2.4; N, 8.3.

### 3.4. [Cu2(Py3As)2Br2] (**2**)

A mixture of CuBr (12 mg, 0.083 mmol) and Py_3_As (26 mg, 0.084 mmol) in CH_3_CN (1 mL) was stirred at room temperature for 10 min. The formed precipitate was centrifuged and dried in vacuum. White powder. Yield: 69 mg (92%). Single crystals of **2** were grown by a diffusion of Et_2_O vapor into an CH_3_CN solution for overnight. ^1^H NMR (500.13 MHz, CDCl_3_, ppm), δ: 9.07 (d, *J* = 4.8 Hz, 6H, H^6^ in Py), 7.92–7.86 (m, 6H, H^4^ in Py), 7.71 (t, *J* = 7.9 Hz, 6H, H^5^ in Py), 7.38–7.32 (m, 6H, H^3^ in Py). FT-IR (KBr, cm^–1^): 405 (m), 417 (m), 459 (w), 474 (m), 490 (s), 619 (w), 637 (m), 671 (w), 696 (w), 733 (w), 743 (w), 766 (vs), 775 (vs), 889 (vw), 966 (w), 989 (m), 1007 (m), 1045 (m), 1082 (w), 1105 (w), 1121 (w), 1153 (m), 1233 (vw), 1277 (w), 1414 (s), 1427 (s), 1449 (vs), 1558 (m), 1578 (s), 1638 (vw), 2953 (w), 2978 (w), 3032 (m), 3057 (w). Calculated for C_30_H_24_As_2_Cu_2_Br_2_N_6_ (905.29): C, 39.8; H, 2.7; N, 9.3. Found: C, 39.7; H, 2.8; N, 9.3.

### 3.5. [Ag@Ag_4_(Py_3_As)_4_](CIO_4_)_5_·3H_2_O (**3**·3H_2_O)

A mixture of AgClO_4_ (20.5 mg, 0.099 mmol) and Py_3_As (25 mg, 0.081 mmol) in CH_3_CN (1 mL) was stirred at room temperature for 10 min. To the resulting solution, diethyl ether (1 mL) was then added, and the precipitate formed was centrifuged and dried in vacuum. White powder. Yield: 156 mg (83%). Single crystals of **3**·3H_2_O were grown by slow evaporation of water solution for few days. ^1^H NMR (500.13 MHz, CD_3_CN, ppm), δ: 8.27–8.21 (m, 12H, H^6^ in Py), 7.89 (t, J = 7.6 Hz, 12H, H^4^ in Py), 7.40 (t, J = 6.5 Hz, 12H, H^5^ in Py), 7.02 (d, J = 7.8 Hz, 12H, H^3^ in Py). FT-IR (KBr, cm^–1^): 405 (m), 467 (s), 507 (m), 621 (vs), 665 (m), 702 (m), 758 (s), 903 (m), 928 (m), 1005 (s), 1047 (vs), 1082 (vs), 1097 (vs), 1165 (m), 1246 (vw), 1287 (w), 1427 (s), 1452 (s), 1558 (m), 1578 (s), 1630 (w), 2995 (w), 3084 (m), 3610 (w). Calculated for C_60_H_54_As_4_Ag_5_N_12_Cl_5_O_23_ (2327.43): C, 31.0; H, 2.3; N, 7.2. Found: C, 30.9; H, 2.5; N, 7.2.

### 3.6. [Ag_2_(Py_2_AsPh)_2_(MeCN)_2_](ClO_4_)_2_·CH_3_CN (**4**·CH_3_CN)

A mixture of AgClO_4_ (15 mg, 0.073 mmol) and bis(2-pyridyl)phenylarsine (25 mg, 0.081 mmol) in CH_3_CN (1 mL) was stirred at room temperature for 10 min. To a resulting solution, diethyl ether (1 mL) was then added, and the precipitate formed was centrifuged and dried in vacuum. White powder. Yield: 72 mg (85%). Single crystals of **4**·CH_3_CN were grown by a diffusion of Et_2_O vapor into an CH_3_CN solution for overnight. ^1^H NMR (500.13 MHz, DMSO-d^6^, ppm), δ: 8.73 (d, *J* = 5.0 Hz, 4H, H^6^ in Py), 7.87 (t, *J* = 7.8 Hz, 4H, H^4^ in Py), 7.56–7.46 (m, 14H, H^5^ in Py, *o*-H, *m*-H and *p*-H in Ph), 7.43 (d, *J* = 7.9 Hz, 4H, H^3^ in Py). FT-IR (KBr, cm^–1^): 405 (w), 469 (m), 488 (m), 623 (s), 696 (m), 743 (s), 764 (m), 926 (w), 989 (m), 1001 (m), 1049 (s), 1099 (vs), 1161 (m), 1236 (vw), 1287 (w), 1425 (s), 1437 (m), 1454 (s), 1483 (w), 1560 (m), 1580 (m), 1636 (w), 1973 (vw), 2251 (w), 2268 (w), 3059 (w). Calculated for C_38_H_35_As_2_Ag_2_N_7_Cl_2_O_8_ (1154.21): C, 39.5; H, 3.1; N, 8.5. Found: C, 39.5; H, 3.0; N, 8.5. 

### 3.7. X-ray Crystallography

The data were collected on a Bruker Kappa Apex II CCD diffractometer using φ, ω-scans of narrow (0.5°) frames with Mo Kα radiation (λ = 0.71073 Å) and a graphite monochromator. The structures were solved by direct methods SHELXL97 and refined by a full matrix least-squares anisotropic-isotropic (for H atoms) procedure using the SHELXL-2018/3 programs set [64]. Absorption corrections were applied using the empirical multiscan method with the SADABS program [65]. The positions of the hydrogen atoms were calculated with the riding model. Free solvent accessible volume in compound **3** derived from PLATON routine analysis was found to be 8.5% (1001.0 Å^3^). This volume is occupied by H_2_O molecules, but this structure is based on very weak data (a better dataset cannot be obtained). Therefore, we employed the PLATON/SQUEEZE procedure to calculate the contribution to the diffraction from H_2_O molecules and thereby produced a set of H_2_O-free diffraction intensities. The final formula of **3**, C_60_H_48_Ag_5_As_4_N_12_Cl_5_O_10_, was derived from the SQUEEZE results. The crystallographic data and details of the structure refinements are summarized in Table S1.

CCDC 2090740, 2090741, 2126017 and 2126016 contain the supplementary crystallographic data for this paper. These data can be obtained free of charge from The Cambridge Crystallographic Data Center at https://www.ccdc.cam.ac.uk/structures/ (accessed on 1 July 2022). 

## 4. Conclusions

In conclusion, the reactions of tris(2-pyridyl)arsine (Py_3_As), an earlier unexplored ligand, with CuI, CuBr, and AgClO_4_ have been studied. The interaction with CuI features a remarkable solvent-directing effect on the product structure. This reaction in CH_2_Cl_2_ results in the crystallization of mononuclear scorpionate [Cu(Py_3_As)I]∙CH_2_Cl_2_, whilst MeCN favors the selective formation of the dimer [Cu_2_(Py_3_As)_2_I_2_]. Noteworthy, scorpionate [Cu(Py_3_As)I]∙CH_2_Cl_2_, when fumed with MeCN vapor, easily (r.t., 1 h) and quantitatively dimerizes into [Cu_2_(Py_3_As)_2_I_2_]. On the contrary, the treatment of Py_3_As with CuBr, regardless of solvent nature, affords the dimer [Cu_2_(Py_3_As)_2_Br_2_] only. At ambient temperature, the above Cu(I) complexes manifest visible photoluminescence with noticeably short decay times (0.8–1.9 μs) and moderate quantum yields (10–14%). Taking into account the results of DFT computations and temperature-dependent photophysical measurements, the emission observed has been tentatively assigned to the thermally activated delayed fluorescence of (M + X) LCT kind (M = Cu, L = Py_3_As; X = halogen).

Complexation of Py_3_As with AgClO_4_ results in the assembly of complex [Ag@Ag_4_(Py_3_As)_4_](CIO_4_)_5_, containing Ag-centered tetrahedron Ag@Ag_4_ supported by four Py_3_As ligands. According to QTAIM analysis of this cluster, argentophilic interactions between its central and peripheral Ag(I) cations are observed.

To sum up, our findings reveal that the coordination chemistry of Py_3_As in some cases, e.g., in the reactions with Cu halides, may differ from that of Py_3_P. Moreover, the Py_3_As-derived Cu(I) complexes demonstrate higher emission rates (k_r_) at 298 K compared to the similar Py_3_P-based analogs, thus highlighting the prospects of employing the arsine ligands for the design of short-lived TADF materials.

## Figures and Tables

**Table 1:** Selected bond lengths (Å) and angles (°) for **1**·CH_2_Cl_2_, **2**, **3**·3H_2_O and **4**·CH_3_CN.

1·CH_2_Cl_2_		2	
Cu–N2	2.046(3)	Cu–As	2.3207(5)
Cu–N1	2.042(2)	Cu–N1′	2.051(3)
Cu–N1′	2.042(2)	Cu–N2′	2.066(2)
Cu–I	2.5003(6)	Cu–Br	2.4242(6)
Symmetry code: (′) x, y, z–1/2.		Symmetry code: (′) 1–x, 1–y, 1–z.	
**3**·3H_2_O		**4**·CH_3_CN	
Ag1∙∙∙Ag2	3.2413(14)	Ag∙∙∙Ag′	3.2206(4)
Ag1∙∙∙Ag3	3.2360(14)	Ag–As′	2.4746(3)
Ag1∙∙∙Ag4	3.2264(14)	Ag–N1	2.347(2)
Ag1∙∙∙Ag5	3.3016(14)	Ag–N2	2.361(2)
Ag1–As1	2.5780(15)	Ag–N3	2.371(3)
Ag1–As2	2.5869(16)	Symmetry code: (′) 1–x, 2–y, 1–z.	
Ag1–As3	2.5900(16)		
Ag1–As4	2.6041(15)		
Ag–N	2.260(13)–2.343(11)		

**Table 2:** Values of the density of all electrons – ρ(**r**), Laplacian of electron density – ∇^2^ρ(**r**) and appropriate λ_2_ eigenvalues, energy density – H_b_, potential energy density – V(**r**), Lagrangian kinetic energy – G(**r**), and electron localization function – ELF at the bond critical points (3, −1), corresponding to Ag(I)···Ag(I) interactions in **3** and **4**.

Ag···Ag Contact	ρ(r)	∇^2^ρ(r)	λ_2_	H_b_	V(r)	G(r)	ELF
**Complex 3**							
3.241 Å	0.019 a.u.	0.030 a.u.	−0.019 a.u.	−0.003 a.u.	−0.013 a.u.	0.010 a.u.	0.125 a.u.
3.236 Å	0.019 a.u.	0.029 a.u.	−0.019 a.u.	−0.003 a.u.	−0.013 a.u.	0.010 a.u.	0.129 a.u.
3.226 Å	0.020 a.u.	0.030 a.u.	−0.020 a.u.	−0.003 a.u.	−0.014 a.u.	0.011 a.u.	0.129 a.u.
3.302 Å	0.017 a.u.	0.029 a.u.	−0.017 a.u.	−0.003 a.u.	−0.012 a.u.	0.009 a.u.	0.112 a.u.
3.249 Å	0.019 a.u.	0.029 a.u.	−0.019 a.u.	−0.003 a.u.	−0.013 a.u.	0.010 a.u.	0.124 a.u.
3.252 Å	0.019 a.u.	0.029 a.u.	−0.019 a.u.	−0.003 a.u.	−0.013 a.u.	0.010 a.u.	0.124 a.u.
3.281 Å	0.018 a.u.	0.029 a.u.	−0.018 a.u.	−0.002 a.u.	−0.012 a.u.	0.010 a.u.	0.117 a.u.
3.209 Å	0.020 a.u.	0.030 a.u.	−0.020 a.u.	−0.003 a.u.	−0.014 a.u.	0.011 a.u.	0.136 a.u.
3.222 Å	0.020 a.u.	0.029 a.u.	−0.020 a.u.	−0.003 a.u.	−0.014 a.u.	0.011 a.u.	0.134 a.u.
3.214 Å	0.020 a.u.	0.030 a.u.	−0.020 a.u.	−0.003 a.u.	−0.014 a.u.	0.011 a.u.	0.134 a.u.
3.247 Å	0.019 a.u.	0.030 a.u.	−0.019 a.u.	−0.003 a.u.	−0.013 a.u.	0.010 a.u.	0.125 a.u.
3.313 Å	0.017 a.u.	0.029 a.u.	−0.017 a.u.	−0.002 a.u.	−0.011 a.u.	0.009 a.u.	0.110 a.u.
**Complex 4**							
3.221 Å	0.021 a.u.	0.028 a.u.	−0.021 a.u.	−0.003 a.u.	-0.014 a.u.	0.011 a.u.	0.156 a.u.

**Table 3:** Luminescent characteristics of Cu(I) complexes **1**, **1a** and **2**.

Complex	λ_em_, nm		PL Lifetime, μs		PLQY, % [298 K]
	298 K	77 K	298 K	77 K	
1·CH_2_Cl_2_	605	625	1.9	25	10 ^a^
**1a**	511	-	0.8	-	14 ^b^
**2**	510	520	0.9	23	12 ^b^

^a^ λ_ex_ = 500 nm, ^b^ λ_ex_ = 390 nm.

## Supplementary Materials

The following supporting information can be downloaded at: https://www.mdpi.com/article/10.3390/molecules27186059/s1, Table S1. X-ray crystallographic data for **1**·CH_2_Cl_2_, **2**, **3**·3H_2_O and **4**·CH_3_CN; Figure S1. Experimental and simulated PXRD patterns of an as-synthesized sample of **1**·CH_2_Cl_2_; Figure S2. Experimental and simulated PXRD patterns of an as-synthesized sample of **2**; Figure S3. ^1^H NMR spectrum of **1**·CH_2_Cl_2_ (CDCl_3_, 25 °C); Figure S4. ^1^H NMR spectrum of **1a** (CD_3_CN, 25 °C); Figure S5. ^1^H NMR spectrum of **2** (CDCl_3_, 25 °C); Figure S6. ^1^H NMR spectrum of **3**·3H_2_O (CD_3_CN, 25 °C); Figure S7. ^1^H NMR spectrum of **4**·CH_3_CN (DMSO-d^6^, 25 °C); Figure S8. FT-IR spectra for the complexes **1**·CH_2_Cl_2_, **1a**, **2**, **3**·3H_2_O and **4**·CH_3_CN in the 400–3250 cm^–1^ region; Figure S9. TGA&DTG curves for **1**·CH_2_Cl_2_, **2**, **3**·3H_2_O and **4**·CH_3_CN; Figure S10. Gibbs free energies calculated for the equilibria 2 [Cu(Py_3_As)X] ↔ [Cu_2_(Py_3_As)_2_X_2_] (X = Br, I) at PBE0/def2TZVP level; Figure S11. Selected frontier molecular orbitals (isovalue = 0.04) calculated for the optimized S_0_ state geometry of [Cu(AsPy_3_)I] (**1**) at PBE0/def2TZVP level; Figure S12. Selected frontier molecular orbitals (isovalue = 0.04) calculated for the optimized S_0_ state geometry of [Cu_2_(Py_3_As)_2_Br_2_] (**2**) at PBE0/def2TZVP level; Figure S13. The UV-Vis spectrum of [Cu(AsPy_3_)I] (**1**) (CH_2_Cl_2_, 298 K) and absorption patterns (vertical bars) calculated at the TD-PBE0/def2TZVP level; Figure S14. The UV-Vis spectrum of [Cu_2_(Py_3_As)_2_Br_2_] (**2**) (MeCN, 298 K) and absorption patterns (vertical bars) calculated at the TD-PBE0/def2TZVP level; Table S2. Atomic contributions to selected molecular orbitals of [Cu(AsPy_3_)I] (**1**) in the ground state (S_0_) geometry according Mulliken population analysis at PBE0/def2TZVP level; Table S3. Atomic contributions to selected molecular orbitals of [Cu_2_(Py_3_As)_2_Br_2_] (**2**) in the ground state (S_0_) geometry according Mulliken population analysis at PBE0/def2TZVP level; Table S4. Calculated (TD-PBE0/def2-TZVP) energies and characters of the main singlet excitations (*f* > 0.01) of [Cu(AsPy_3_)I] (**1**); Table S5. Calculated (TD-PBE0/def2-TZVP) energies and characters of the main singlet excitations (*f* > 0.01) of [Cu_2_(Py_3_As)_2_Br_2_] (**2**); Figure S15. LSOMO and HSOMO (isovalue = 0.04) calculated for the optimized gas phase T_1_ state geometry of [Cu(AsPy_3_)I] (**1**) at PBE0/def2TZVP level; Figure S16. Temperature dependent excitation spectra of **1**·CH_2_Cl_2_ (a) and **2** (b) recorded at λ_reg_ = 595 and 520 nm, respectively; Figure S17. Red-shifting emission profile of **1**·CH_2_Cl_2_ (left) and **2** (right) upon cooling from 298 to 77 K; Figure S18. PL decay kinetics for **1**·CH_2_Cl_2_ (left) and **2** (right). Citation of reference [66,67,68,69,70,71,72,73,74,75,76,77,78].

## Sample Availability

Samples used are available from the authors. Crystallographic details, ^1^H NMR and FT-IR spectra, TGA&DTG curves, and computation details.
