# Supplementary material for: Tris(2-Pyridyl)Arsine as a New Platform for Design of Luminescent Cu(I) and Ag(I) Complexes
Source: Molecules. 2022 Sep 16;27(18):6059. doi: 10.3390/molecules27186059 (PMC9503387; doi:10.3390/molecules27186059)
Supplement: Supplementary file 1 [file molecules-27-06059-s001.zip › molecules-1904557-supplementary.pdf]

Table of contents

S1 §1. Single crystal X-ray crystallography

S2–3 §2. Powder X-ray diffraction patterns **1**·CH_2Cl_2 and **2**

S3–5 §3. ^{1}H NMR spectra

S6 §4. FT-IR spectra

S6 §5. TGA&DTG curves

S7 §6. Computational details

S7 §6.1. Relative stabilities of complexes [Cu(Py_3As)X] and [$Cu_2(Py_3As)_2X_2$] (X = Br, I)

S8–13 §6.2. Molecular orbital’ calculations

S14 §6.3. QTAIM and ELF calculations

S14–15 §7. Photophysical details

S15 §8. References

§1. Single crystal X-ray crystallography

Table S1. X-Ray crystallographic data for **1**·CH_2Cl_2, **2**, **3**·$3H_2O$ and **4**·CH_3CN.

	**1**·CH_2Cl_2	**2**	**3**·$3H_2O$	**4**·CH_3CN
Crystal data	2090740	2090741	2126017	2126016
Chemical formula	$C_{15}H_{12}AsCuIN_3$	$C_{30}H_{24}As_2Cu_2Br_2N_6$	$C_{60}H_{48}Ag_5As_4N_{12}\cdot5(ClO_4)$	$C_{36}H_{32}Ag_2As_2N_6\cdot2(ClO_4)\cdot2(C_2H_3N)$
M_r	584.56	905.29	2273.38	1195.26
Crystal system, space group	Orthorhombic, *Pbcm*	Monoclinic, $P2_1/n$	Triclinic, *P*1	Monoclinic, $P2_1/c$
Temperature (K)	200	296	200	200
a, *b*, *c* (Å)	8.0576(10), 16.629(2), 14.450(2)	8.8551(8), 11.7729(13), 15.7798(15)	15.5058(13), 22.0968(19), 35.863(3)	14.5721(7), 8.5513(3), 19.9140(8)
β(°)	-	104.157(5)	-	110.961(2)
V (Å^3)	1936.1(4)	1595.1(3)	11720.5(17)	2317.28(17)
Z	4	2	6	2
μ (mm^{-1})	4.70	5.93	3.16	2.44

Crystal size (mm)	0.25 × 0.20 × 0.01	0.15 × 0.08 × 0.04	0.15 × 0.15 × 0.08	0.50 × 0.04 × 0.02
T_{min}, T_{max}	0.653, 0.928	0.776, 0.958	0.810, 0.928	0.715, 0.862
No. of measured, independent and observed [$I > 2\sigma(I)$] reflections	11744, 2320, 1997	15644, 3669, 2627	158498, 41400, 18320	18686, 5315, 4223
R_{int}	0.054	0.053	0.109	0.050
$(\sin \theta/\lambda)_{max}$ (Å^{-1})	0.650	0.650	0.595	0.652
$R[F^2 > 2\sigma(F^2)]$, $wR(F^2)$, S	0.027, 0.070, 1.01	0.032, 0.068, 1.00	0.068, 0.179, 0.90	0.030, 0.069, 1.01
No. of reflections	2320	3669	41400	5315
No. of parameters	123	190	2863	280
$\Delta\rho_{max}$, $\Delta\rho_{min}$ (e Å^{-3})	0.74, −0.53	0.49, −0.42	3.28, −1.72	0.45, −0.43

§2. Powder X-ray diffraction patterns of 1·CH_2Cl_2 and 2

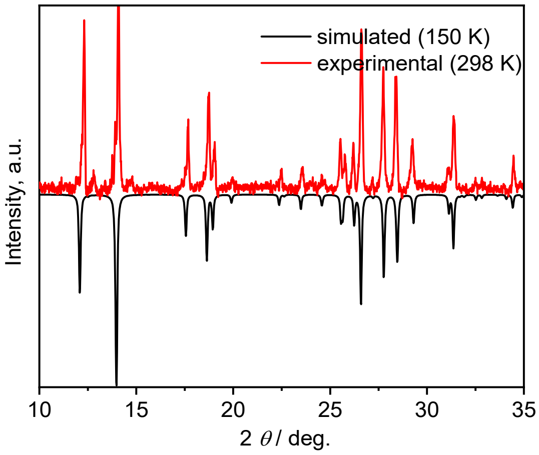

Figure S1. Experimental and simulated PXRD patterns of an as-synthesized sample of **1**·CH_2Cl_2.

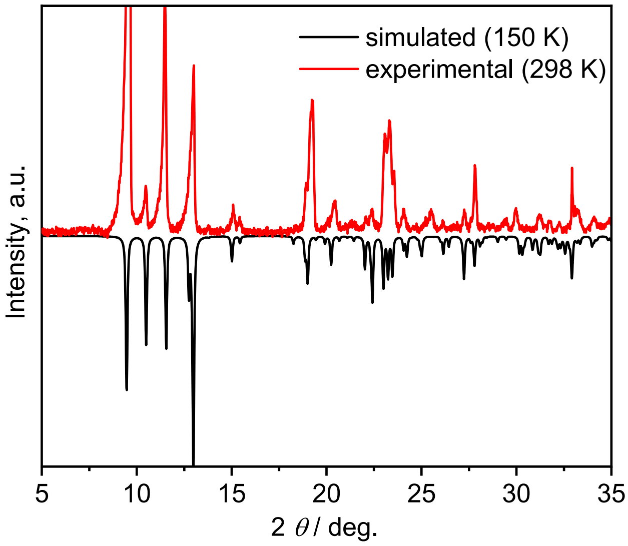

Figure S2. Experimental and simulated PXRD patterns of an as-synthesized sample of **2**.

§3. ^{1}H NMR spectra

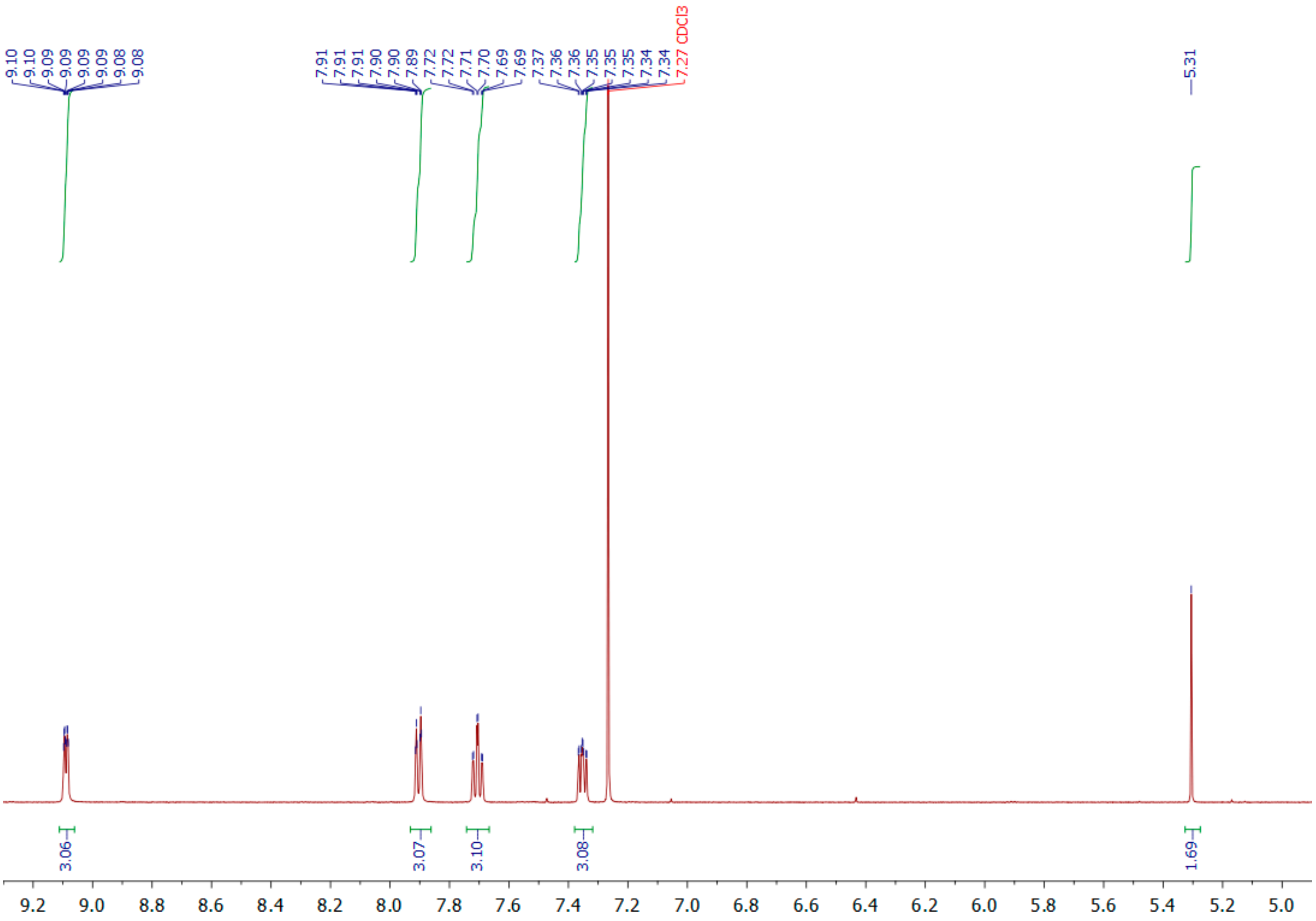

Figure S3. ^{1}H NMR spectrum of **1**·CH_2Cl_2 ($CDCl_3$, 25 °C).

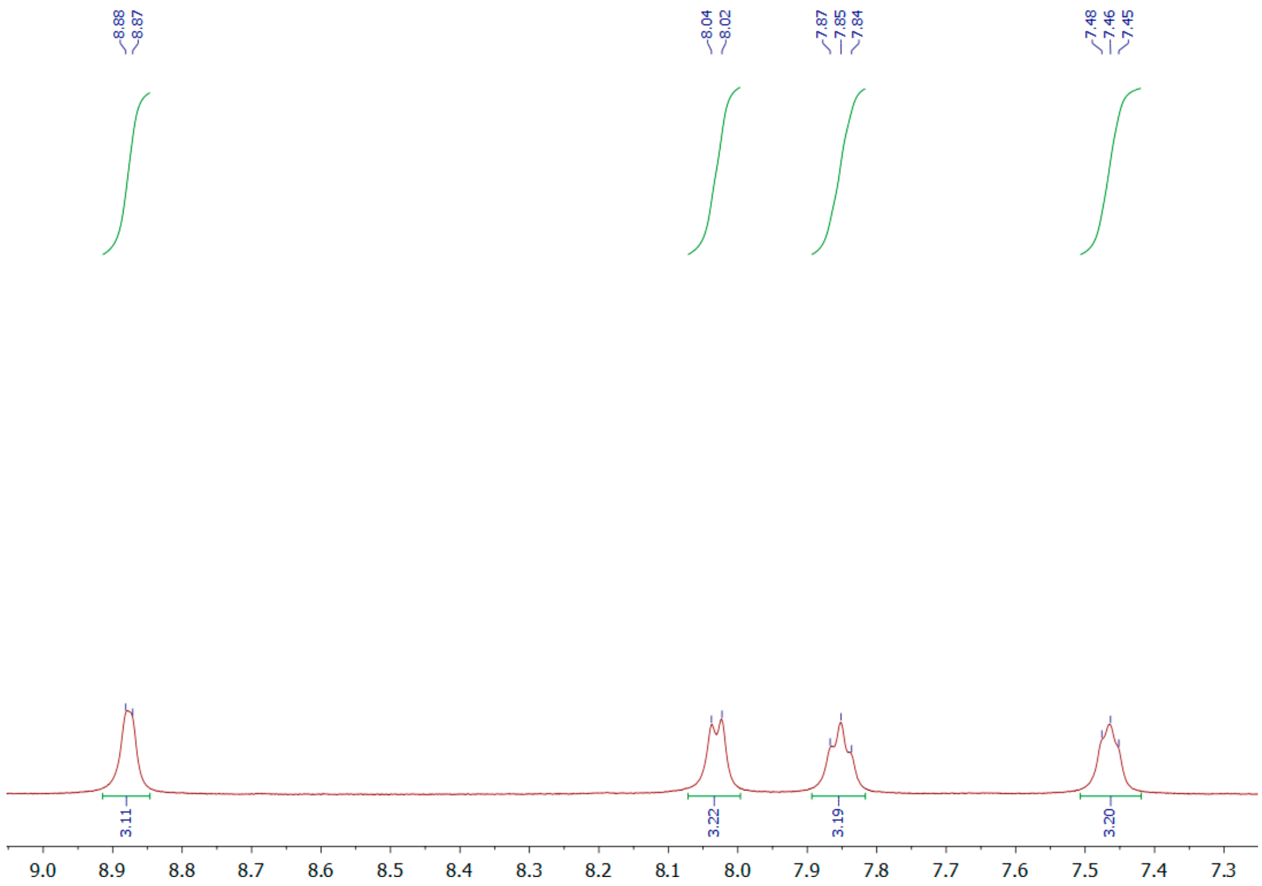

Figure S4. ^{1}H NMR spectrum of **1a** (CD_3CN, 25 °C).

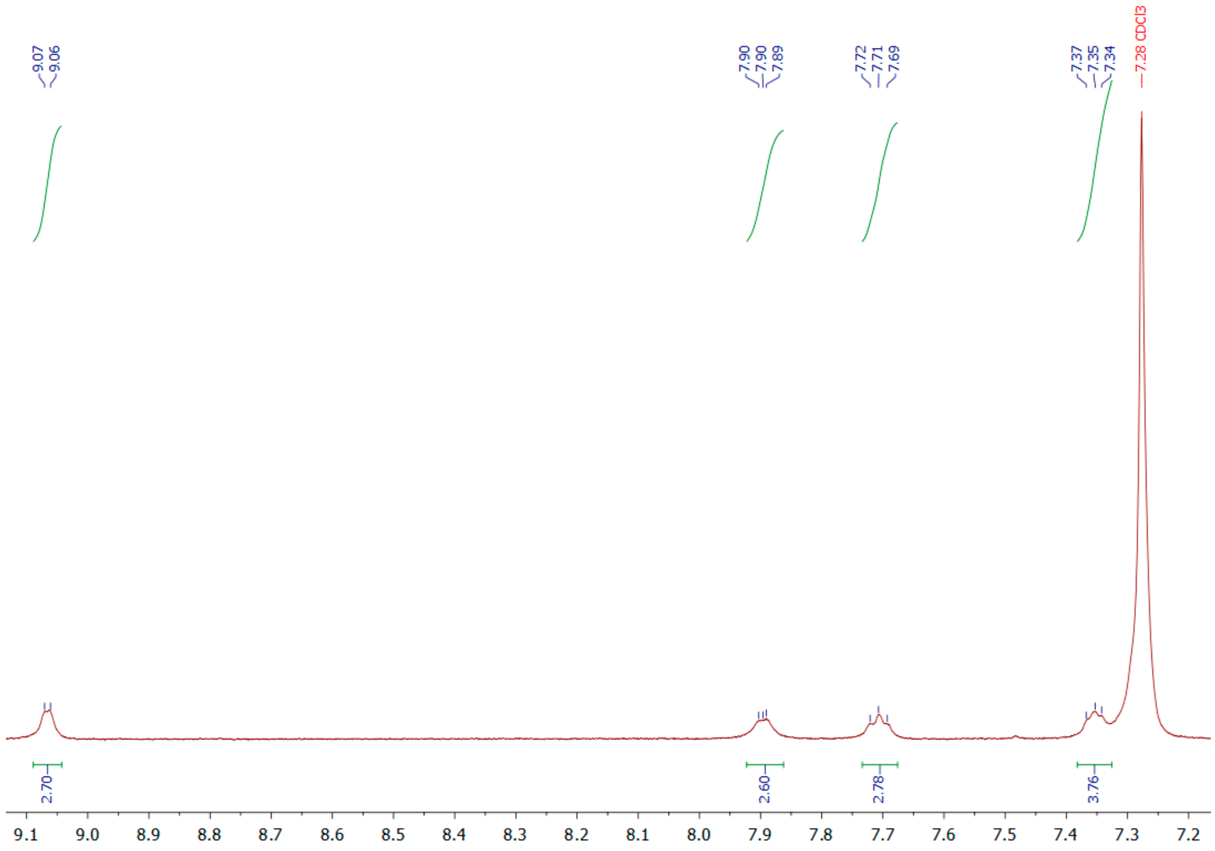

Figure S5. ^{1}H NMR spectrum of **2** ($CDCl_3$, 25 °C).

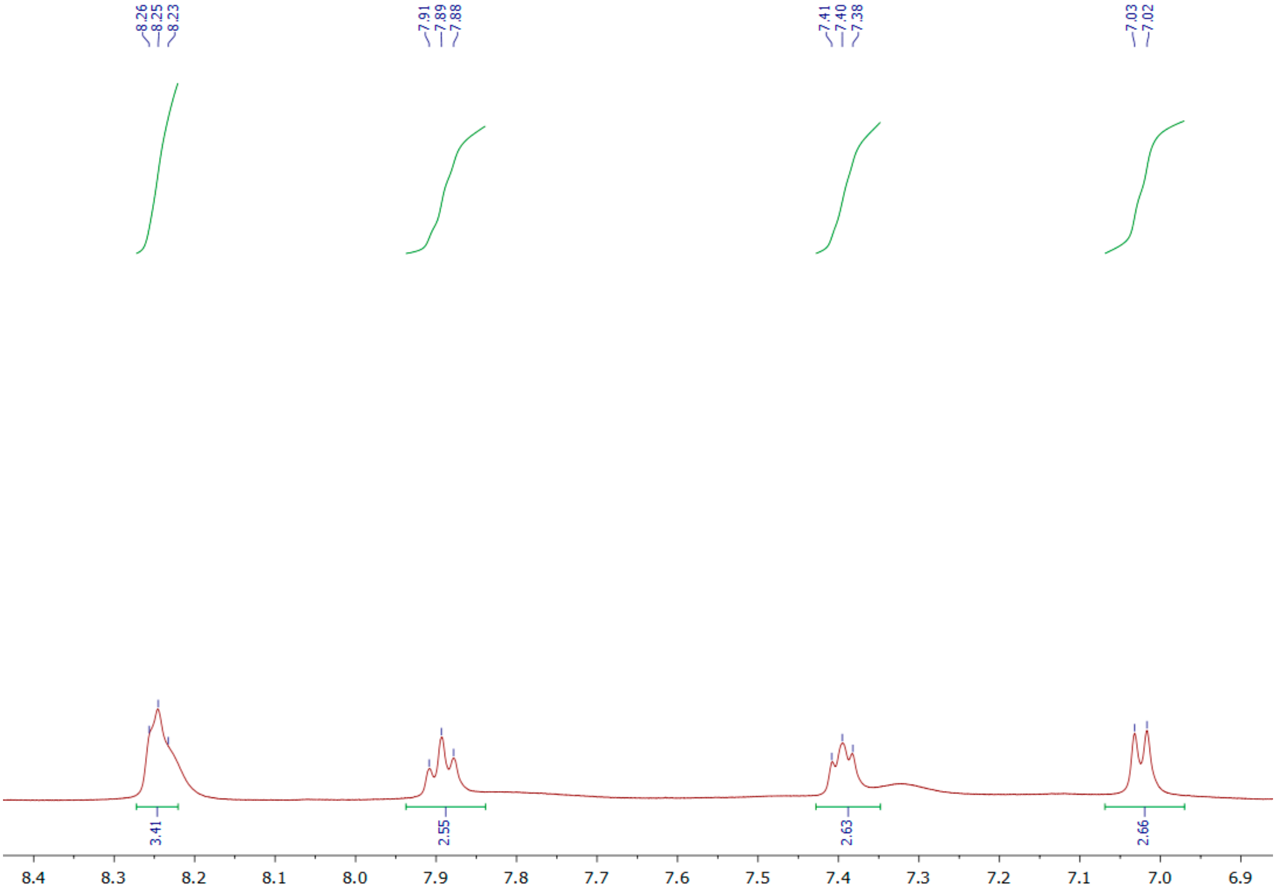

Figure S6. ^{1}H NMR spectrum of **3**·3H_2O (CD_3CN, 25 °C).

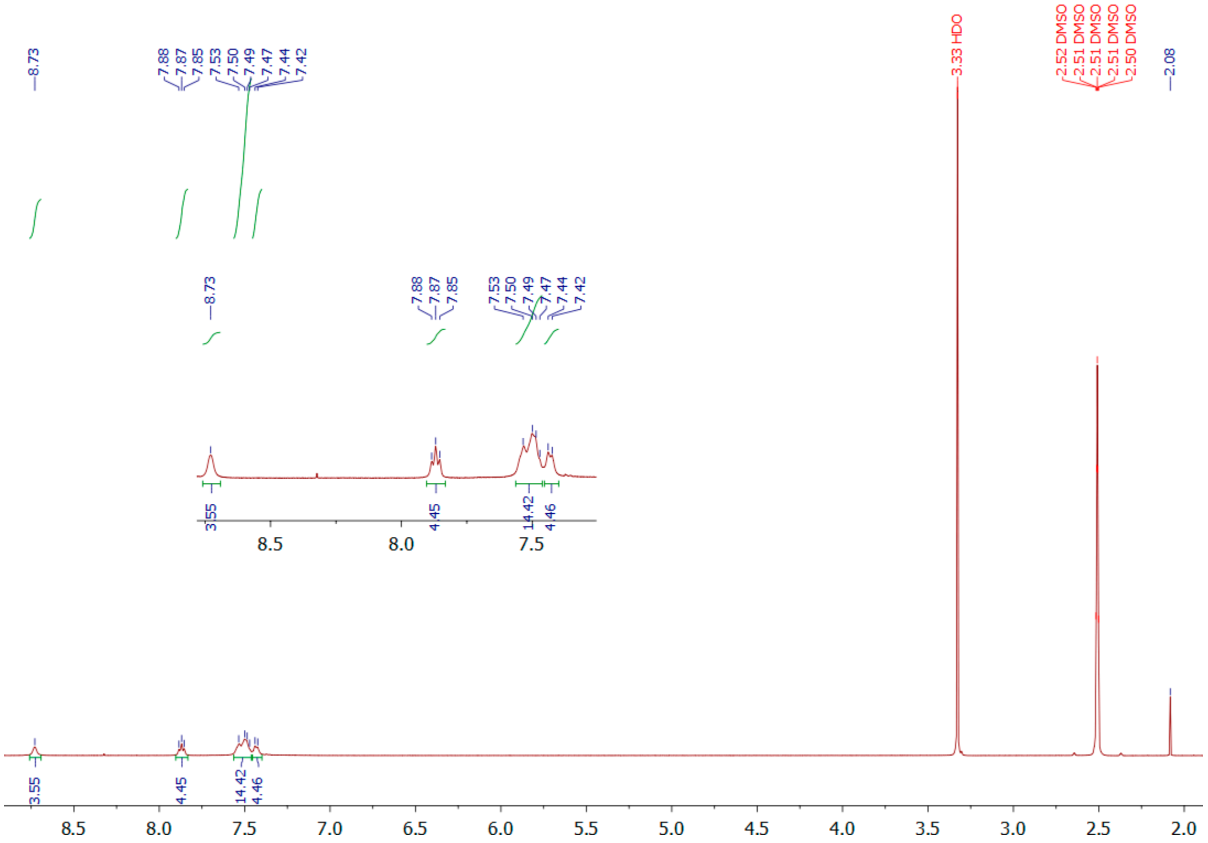

Figure S7. ^{1}H NMR spectrum of **4**·CH_3CN (DMSO-d^6, 25 °C).

§4. FT-IR spectra

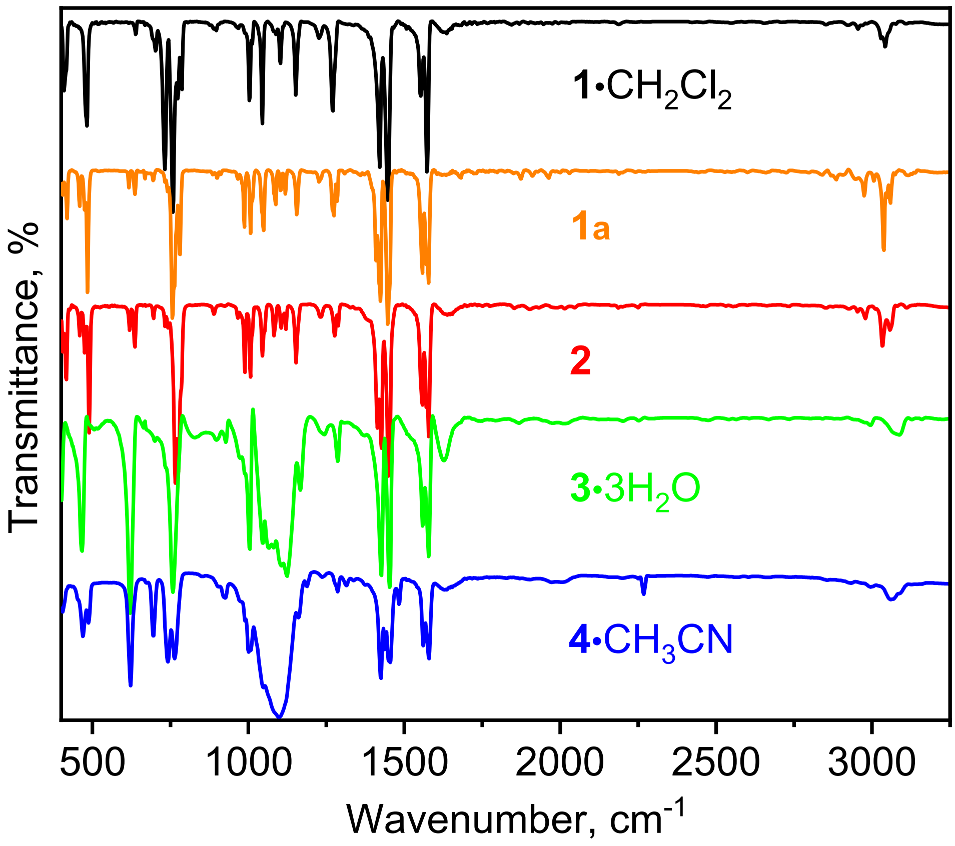

Figure S8. FT-IR spectra for the complexes **1**·CH_2Cl_2, **1a**, **2**, **3**·$3H_2O$ and **4**·CH_3CN in the 400–3250 cm^{-1} region.

§5. TGA&DTG curves

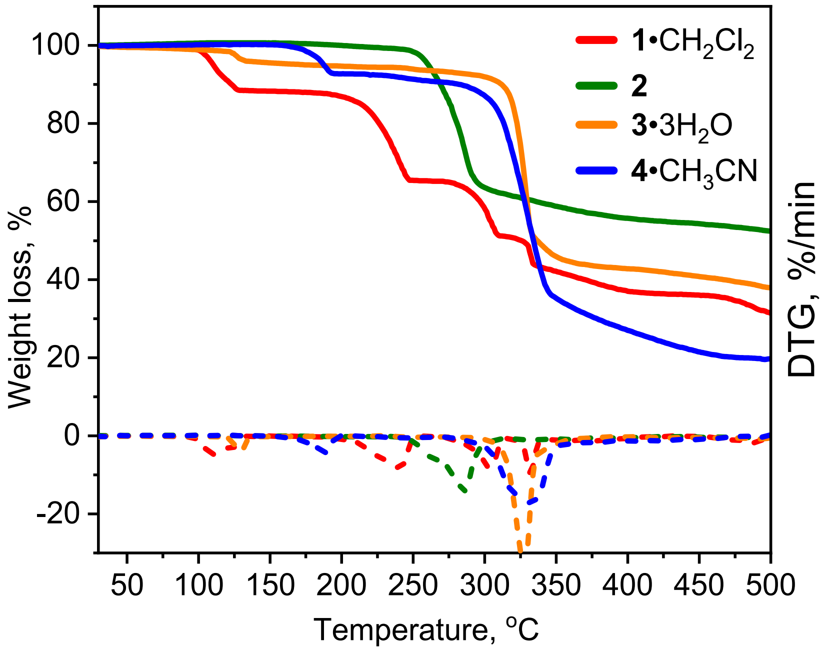

Figure S9. TGA&DTG curves for **1**·CH_2Cl_2, **2**, **3**·$3H_2O$ and **4**·CH_3CN.

§6. Computational details

DFT and TD-DFT computations were performed using Gaussian 09 program.[1] The structures of the S_0 were fully optimized using PBE0[2] hybrid functionals coupled with the def2-TZVP basis set.[3] There are no imaginary frequencies were found in all optimized structures. The absorption spectrum was computed using time-dependent DFT (TD-DFT)[4] calculations with the optimized ground state (S_0) geometry.

§6.1. Relative stabilities of complexes [Cu(Py_3As)X] and [Cu_2(Py_3As)$_2$$X_2$] (X = Br, I)

To estimate the relative stabilities of scorpionate [Cu(Py_3As)X] and dimeric [Cu_2(Py_3As)$_2$$X_2$] complexes (X = Br, I), their geometries were optimized at PBE0/def2TZVP level of the theory. The Gibbs free energies of these complexes were calculated at the same level. The results of calculations are outlined in Figure S1.

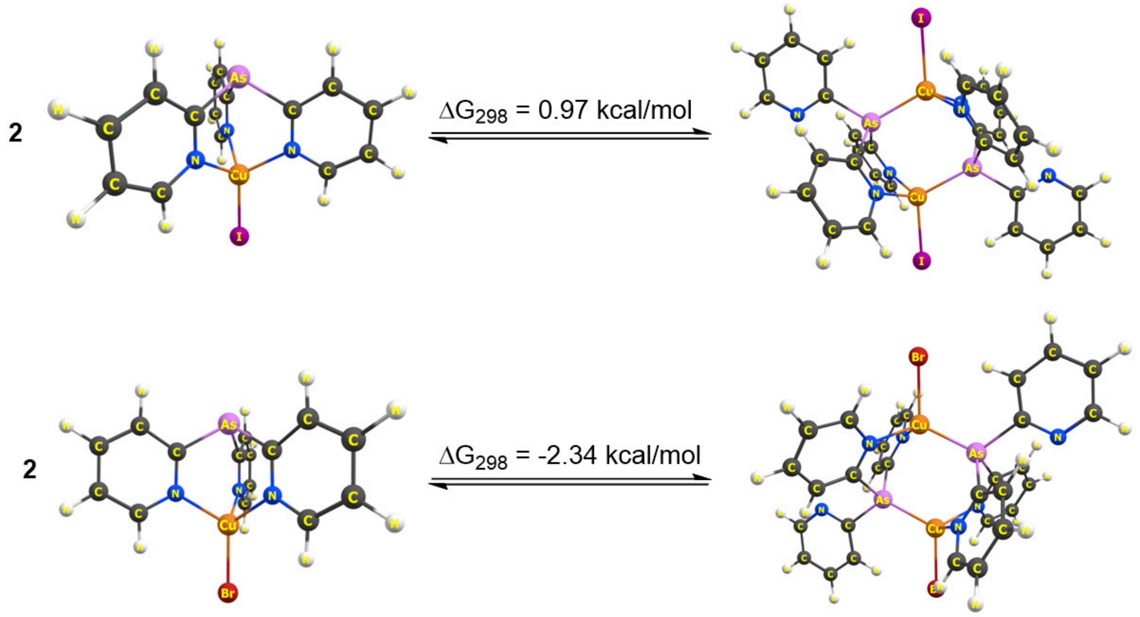

Figure S10. Gibbs free energies calculated for the equilibria 2 [Cu(Py_3As)X] ↔ [Cu_2(Py_3As)$_2$$X_2$] (X = Br, I) at PBE0/def2TZVP level.

§6.2. Molecular orbital' calculations

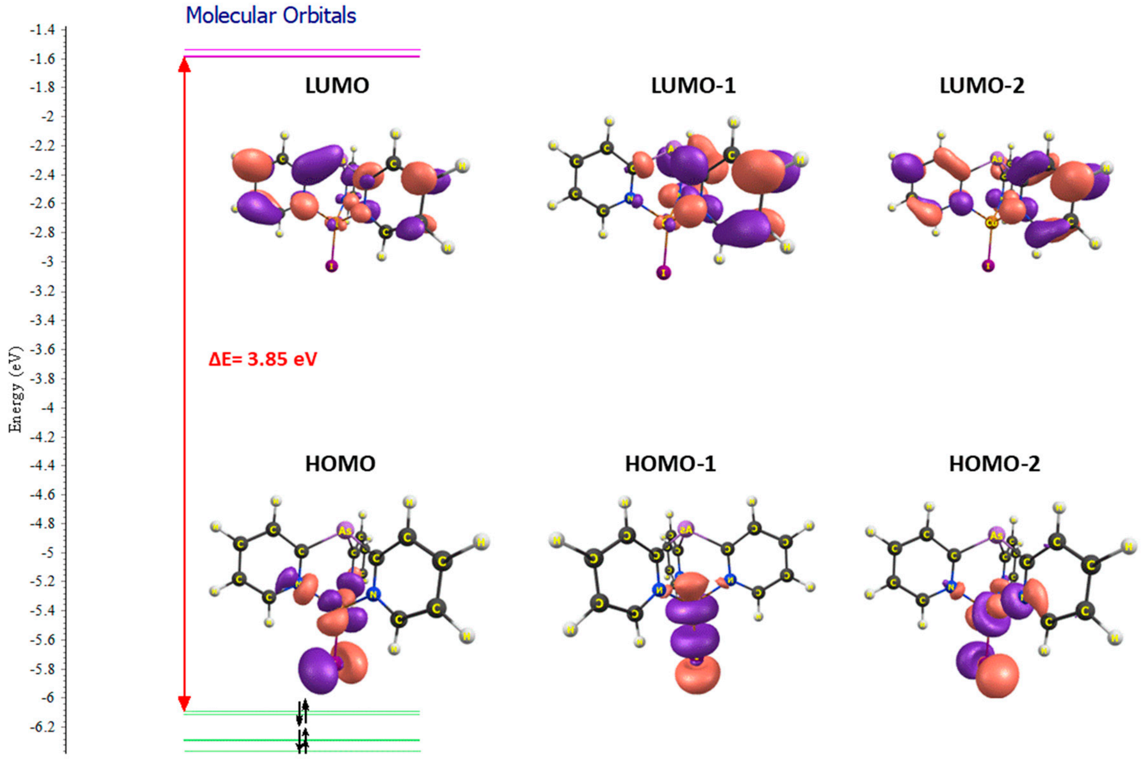

Figure S11. Selected frontier molecular orbitals (isovalue = 0.04) calculated for the optimized S_0 state geometry of [Cu(AsPy$_3$)I] (**1**) at PBE0/def2TZVP level.

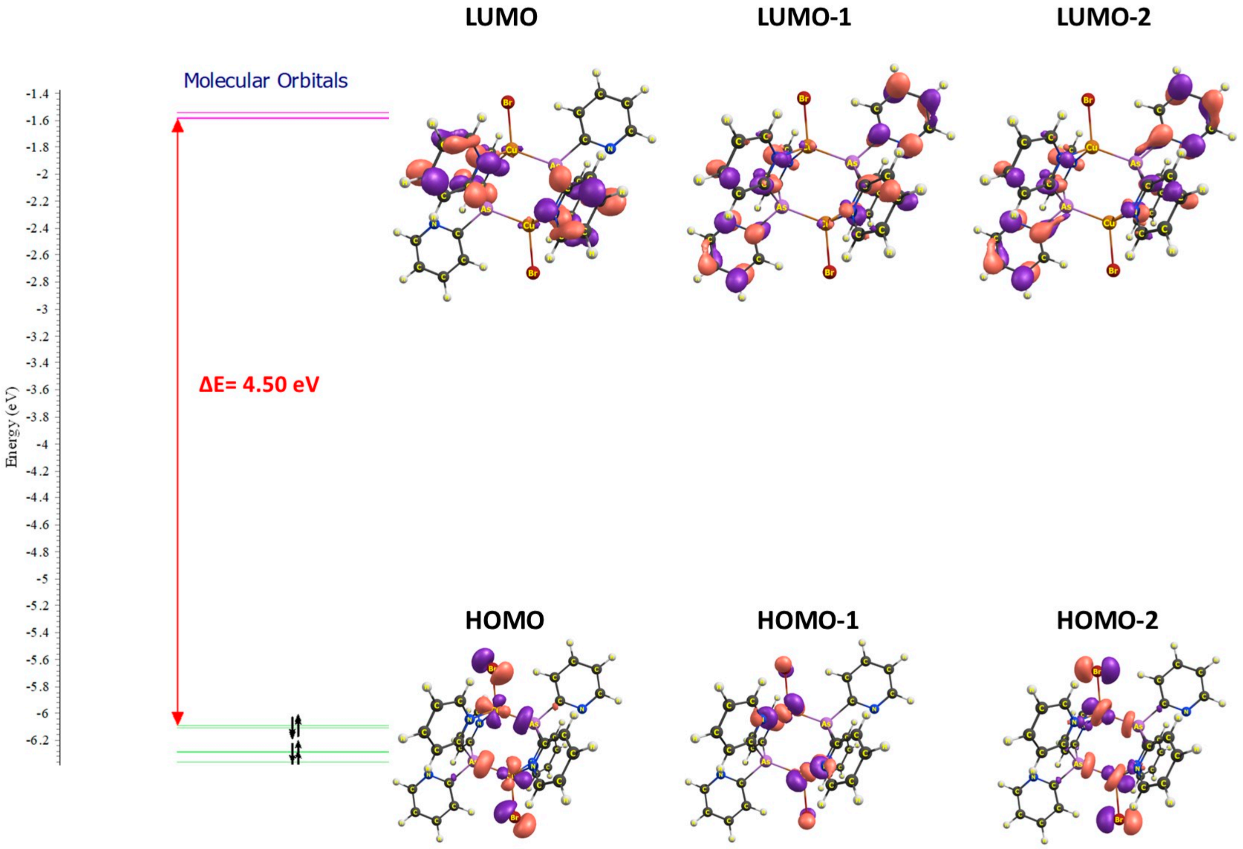

Figure S12. Selected frontier molecular orbitals (isovalue = 0.04) calculated for the optimized S_0 state geometry of [Cu$_2$(Py$_3$As)$_2$Br$_2$] (**2**) at PBE0/def2TZVP level.

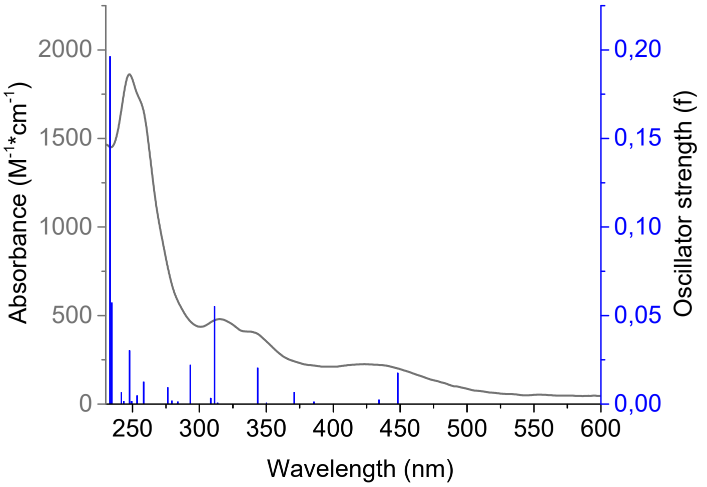

Figure S13. The UV-Vis spectrum of [Cu(AsPy$_3$)I] (**1**) (CH_2Cl_2, 298 K) and absorption patterns (vertical bars) calculated at the TD-PBE0/def2TZVP level.

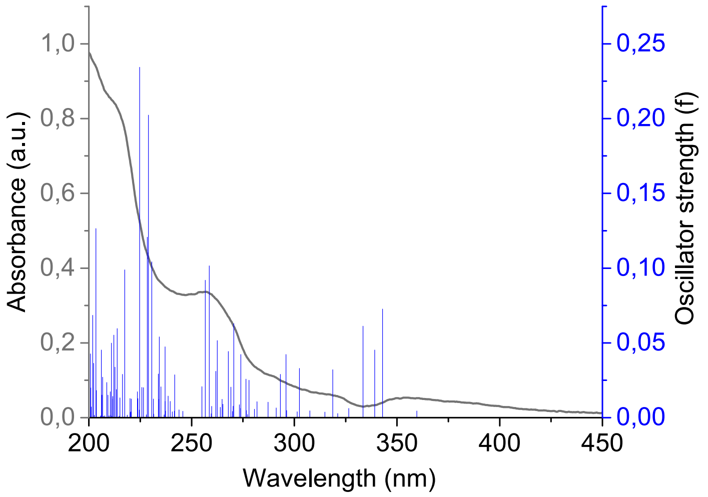

Figure S14. The UV-Vis spectrum of [Cu$_2$(Py$_3$As)$_2$Br$_2$] (**2**) (MeCN, 298 K) and absorption patterns (vertical bars) calculated at the TD-PBE0/def2TZVP level.

Table S2. Atomic contributions to selected molecular orbitals of [Cu(AsPy$_3$)I] (**1**) in the ground state (S_0) geometry according Mulliken population analysis at PBE0/def2TZVP level.

MO	Energy, eV	Contribution, %		
		**Cu**	**I**	**Py$_3$As**
**LUMO+2**	-1,13853	0	0	100
**LUMO+1**	-1,64168	2	0	98
**LUMO**	-1,64195	2	0	98
**HOMO**	-5,48858	58	24	18
**HOMO-1**	-5,48885	58	24	18
**HOMO-2**	-5,93104	63	31	6
**HOMO-3**	-6,33459	63	29	9
**HOMO-4**	-6,33486	63	29	9
**HOMO-5**	-6,96671	47	47	6
**HOMO-6**	-6,96726	47	47	6

**HOMO-7**	-7,59639	3.3	0.04	96.3
**HOMO-8**	-7,8198	43	55	2
**HOMO-9**	-7,91667	0	0	100
**HOMO-10**	-8,01844	4	0	95
**HOMO-11**	-8,01844	4	0	95

Table S3. Atomic contributions to selected molecular orbitals of $[Cu_2(Py_3As)_2Br_2]$ (**2**) in the ground state (S_0) geometry according Mulliken population analysis at PBE0/def2TZVP level.

MO	Energy, eV	Contribution, %	
		**CuBr**	**L**
**LUMO+2**	-1,54127	2	98
**LUMO+1**	-1,58508	1	99
**LUMO**	-1,58834	3	97
**HOMO**	-6,08941	69	31
**HOMO-1**	-6,10683	70	30
**HOMO-2**	-6,28289	74	26
**HOMO-3**	-6,29078	77	23
**HOMO-4**	-6,36126	89	11
**HOMO-5**	-6,46901	85	15
**HOMO-6**	-6,91338	93	7
**HOMO-7**	-6,97814	90	10
**HOMO-8**	-6,98903	87	13
**HOMO-9**	-7,02359	85	15
**HOMO-10**	-7,54088	48	52
**HOMO-11**	-7,54578	48	52

Table S4. Calculated (TD-PBE0/def2-TZVP) energies and characters of the main singlet excitations (f > 0.01) of $[Cu(AsPy_3)I]$ (**1**).

E, eV	λ, nm	f [(a)]	Transitions (main contributions)	Character[(b)]
2.766	448.18	0.018	H-1 -> L+1 (48%)<br>HOMO -> LUMO (48%)	(CuI)LCT
2.767	448.16	0.018	HOMO -> L+1 (48%)<br>H-1 -> LUMO (48%)	(CuI)LCT
3.609	343.53	0.021	H-4 -> LUMO (26%)<br>H-3 -> L+1 (25%)<br>H-4 -> L+1 (20%)<br>H-3 -> LUMO (20%)	(CuI)LCT
3.609	343.53	0.020	H-4 -> L+1 (26%)<br>H-3 -> LUMO (25%)<br>H-3 -> L+1 (21%)<br>H-4 -> LUMO (20%)	(CuI)LCT
3.982	311.33	0.055	H-4 -> L+1 (26%)<br>H-3 -> LUMO (26%)<br>H-3 -> L+1 (21%)<br>H-4 -> LUMO (21%)	(CuI)LCT
4.229	293.16	0.022	H-3 -> L+2 (95%)	(CuI)LCT

			H-6 -> L+2 (4%)	
4.299	293.15	0.022	H-4 -> L+2 (95%)	(CuI)LCT
5.314	233.32	0.196	H-9 -> LUMO (24%)<br>H-8 -> L+1 (14%)<br>H-11 -> LUMO (13%)<br>H-9 -> L+1 (13%)<br>H-10 -> L+1 (13%)	(L)LCT+(CuI)LCT
5.314	233.31	0.196	H-9 -> L+1 (24%)<br>H-8 -> LUMO (14%)<br>H-11 -> L+1 (13%)<br>H-10 -> LUMO (13%)<br>H-9 -> LUMO (13%)	(L)LCT+(CuI)LCT

[(a)] Oscillator strength; [(b)] L is Py_3As.

Table S5. Calculated (TD-PBE0/def2-TZVP) energies and characters of the main singlet excitations (f > 0.01) of $[Cu_2(Py_3As)_2Br_2]$ (**2**).

E, eV	λ, nm	f [(a)]	Transitions (main contributions)	Character[(b)]
3.615	342.94	0.073	HOMO -> LUMO (50%)<br>H-1 -> L+2 (30%)	(CuBr)LCT
3.718	333.472	0.061	HOMO -> L+2 (88%)	(CuBr)LCT
4.525	273.99	0.042	H-2 -> L+7 (61%)<br>H-4 -> L+6 (20%)	(CuBr)LCT
4.582	270.59	0.063	H-6 -> L+2 (31%)<br>H-7 -> L+1 (19%)<br>H-3 -> L+8 (17%)	(CuBr)LCT
4.607	269.13	0.021	H-7 -> L+1 (67%)<br>H-6 -> L+2 (18%)	(CuBr)LCT
4.794	258.62	0.102	HOMO -> L+12 (52%)<br>H-8 -> L+3 (23%)<br>H-9 -> LUMO (14%)	(CuBr)LCT
4.831	256.66	0.092	H-8 -> L+3 (44%)<br>HOMO -> L+12 (26%)	(CuBr)LCT
5.414	228.99	0.0202	H-15 -> LUMO (39%)<br>H-16 -> L+3 (13%)<br>H-8 -> L+8 (12%)	(CuBr)LCT
5.427	228.48	0.121	H-13 -> L+2 (17%)<br>H-19 -> L+1 (15%)<br>H-16 -> L+1 (11%)<br>H-12 -> L+1 (10%)<br>H-18 -> L+2 (10%)	(CuBr)LCT
5.517	224.72	0.234	H-16 -> L+1 (25%)<br>H-15 -> L+2 (16%)<br>H-19 -> L+1 (14%)<br>H-18 -> L+2 (13%)	L(L)CT
5.538	223.87	0.013	H-6 -> L+12 (42%)<br>H-12 -> L+3 (17%)<br>H-3 -> L+13 (9%)	(CuBr)LCT+(L)LCT

5.545	223.62	0.017	H-14 -> LUMO (49%)<br>H-11 -> L+3 (19%)	(CuBr)LCT+(L)LCT

[(a)] Oscillator strength; [(b)] L is Py_3As.

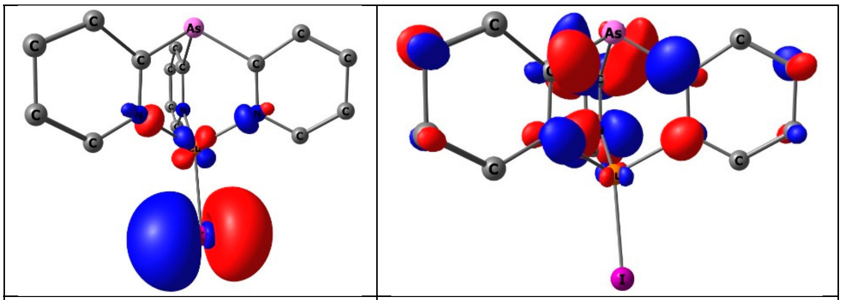

LSOMO (–6.490 eV)	HSOMO (–3.120 eV)

Figure S15. LSOMO and HSOMO (isovalue = 0.04) calculated for the optimized gas phase T_1 state geometry of [Cu($AsPy_3$)I] (**1**) at PBE0/def2TZVP level.

Cartesian coordinates for gas phase S_0 and T_1 state geometries of [Cu($AsPy_3$)I] (**1**) at PBE0/def2TZVP level.

S_0 state				T_1 state			
I	3.346487000	0.000374000	0.000316000	I	3.346487000	0.000374000	0.000316000
As	-2.518224000	-0.000561000	-0.000578000	As	-2.518224000	-0.000561000	-0.000578000
Cu	0.835421000	0.000435000	0.000233000	Cu	0.835421000	0.000435000	0.000233000
N	-0.242601000	-1.613122000	-0.759448000	N	-0.242601000	-1.613122000	-0.759448000
N	-0.243744000	0.148766000	1.776790000	N	-0.243744000	0.148766000	1.776790000
C	0.366450000	-2.685437000	-1.264223000	C	0.366450000	-2.685437000	-1.264223000
H	1.452037000	-2.655197000	-1.249929000	H	1.452037000	-2.655197000	-1.249929000
C	-0.334699000	-3.765931000	-1.773100000	C	-0.334699000	-3.765931000	-1.773100000
H	0.201663000	-4.617116000	-2.173822000	H	0.201663000	-4.617116000	-2.173822000
C	-1.719163000	-3.728332000	-1.755621000	C	-1.719163000	-3.728332000	-1.755621000
H	-2.300891000	-4.556019000	-2.145452000	H	-2.300891000	-4.556019000	-2.145452000
C	-2.351096000	-2.612041000	-1.230269000	C	-2.351096000	-2.612041000	-1.230269000
H	-3.432334000	-2.543982000	-1.198416000	H	-3.432334000	-2.543982000	-1.198416000
C	-1.573863000	-1.570012000	-0.739511000	C	-1.573863000	-1.570012000	-0.739511000
C	-1.574985000	0.144351000	1.728681000	C	-1.574985000	0.144351000	1.728681000
C	-2.352916000	0.240171000	2.876037000	C	-2.352916000	0.240171000	2.876037000
H	-3.434109000	0.233580000	2.800543000	H	-3.434109000	0.233580000	2.800543000
C	-1.721718000	0.343297000	4.105843000	C	-1.721718000	0.343297000	4.105843000
H	-2.303993000	0.419458000	5.017212000	H	-2.303993000	0.419458000	5.017212000
C	-0.337284000	0.347193000	4.147969000	C	-0.337284000	0.347193000	4.147969000
H	0.198507000	0.425885000	5.085797000	H	0.198507000	0.425885000	5.085797000
C	0.364568000	0.247810000	2.958220000	C	0.364568000	0.247810000	2.958220000
H	1.450176000	0.245429000	2.925551000	H	1.450176000	0.245429000	2.925551000
N	-0.243343000	1.464556000	-1.017051000	N	-0.243343000	1.464556000	-1.017051000
C	0.365100000	2.438431000	-1.693089000	C	0.365100000	2.438431000	-1.693089000
H	1.450703000	2.411589000	-1.674161000	H	1.450703000	2.411589000	-1.674161000
C	-0.336648000	3.418995000	-2.374312000	C	-0.336648000	3.418995000	-2.374312000
H	0.199250000	4.192043000	-2.910959000	H	0.199250000	4.192043000	-2.910959000
C	-1.721095000	3.384111000	-2.350641000	C	-1.721095000	3.384111000	-2.350641000
H	-2.303279000	4.135224000	-2.872490000	H	-2.303279000	4.135224000	-2.872490000
C	-2.352392000	2.370326000	-1.646994000	C	-2.352392000	2.370326000	-1.646994000
H	-3.433591000	2.307950000	-1.604127000	H	-3.433591000	2.307950000	-1.604127000
C	-1.574590000	1.424709000	-0.990025000	C	-1.574590000	1.424709000	-0.990025000

Cartesian coordinates for gas phase S_0 state geometries of [$Cu_2(Py_3As)_2Br_2$] (**2**) at PBE0/def2TZVP level.

S_0 state			
Br	-2.474720000	-3.618512000	-0.000006000
As	-2.042576000	0.394624000	0.000001000

Cu	-0.979511000	-1.709962000	-0.000001000
N	-0.398111000	2.052366000	1.548248000
N	-0.398114000	2.052369000	-1.548249000
C	-1.649512000	1.593160000	1.504159000
C	-2.576416000	1.880260000	2.496623000
H	-3.585650000	1.498120000	2.431519000
C	-2.185152000	2.676513000	3.559721000
H	-2.887616000	2.912797000	4.351196000
C	-0.893387000	3.175947000	3.588305000
H	-0.551186000	3.814325000	4.393145000
C	-0.033299000	2.845423000	2.557132000
H	0.982160000	3.225856000	2.505563000
C	-0.033305000	2.845430000	-2.557130000
H	0.982157000	3.225860000	-2.505564000
C	-0.893398000	3.175965000	-3.588295000
H	-0.551198000	3.814342000	-4.393136000
C	-2.185166000	2.676539000	-3.559703000
H	-2.887635000	2.912832000	-4.351171000
C	-2.576428000	1.880283000	-2.496606000
H	-3.585664000	1.498152000	-2.431495000
C	-1.649517000	1.593170000	-1.504151000
C	-4.004899000	0.450074000	0.000001000
C	-4.706261000	-0.748139000	-0.000006000
H	-4.175214000	-1.698002000	-0.000007000
C	-6.093754000	-0.689774000	-0.000009000
H	-6.675675000	-1.604678000	-0.000014000
C	-6.713089000	0.547287000	-0.000002000
H	-7.792907000	0.638365000	-0.000004000
C	-5.917002000	1.685964000	0.000007000
H	-6.366803000	2.675273000	0.000013000
N	-4.590494000	1.646461000	0.000008000
Br	2.474719000	3.618512000	0.000004000
As	2.042576000	-0.394623000	0.000000000
Cu	0.979511000	1.709962000	0.000000000
N	0.398113000	-2.052367000	-1.548248000
N	0.398113000	-2.052367000	1.548249000
C	1.649514000	-1.593162000	-1.504157000
C	2.576420000	-1.880265000	-2.496618000
H	3.585654000	-1.498125000	-2.431512000
C	2.185158000	-2.676519000	-3.559715000
H	2.887624000	-2.912806000	-4.351188000
C	0.893393000	-3.175953000	-3.588302000
H	0.551193000	-3.814332000	-4.393141000
C	0.033303000	-2.845426000	-2.557132000
H	-0.982158000	-3.225858000	-2.505564000
C	0.033303000	-2.845427000	2.557131000
H	-0.982158000	-3.225858000	2.505564000
C	0.893394000	-3.175959000	3.588299000
H	0.551194000	-3.814335000	4.393140000
C	2.185162000	-2.676531000	3.559708000
H	2.887629000	-2.912822000	4.351179000
C	2.576425000	-1.880276000	2.496611000
H	3.585660000	-1.498145000	2.431501000
C	1.649516000	-1.593167000	1.504154000
C	4.004899000	-0.450074000	-0.000002000
C	4.706262000	0.748138000	-0.000011000
H	4.175216000	1.698002000	-0.000017000
C	6.093755000	0.689772000	-0.000014000
H	6.675677000	1.604676000	-0.000021000
C	6.713089000	-0.547290000	-0.000006000
H	7.792906000	-0.638369000	-0.000007000
C	5.917001000	-1.685966000	0.000002000
H	6.366801000	-2.675276000	0.000007000
N	4.590493000	-1.646462000	0.000004000

§6.3. QTAIM and ELF calculations

The QTAIM and ELF calculations of cations of **3** and **4**, $[Ag@Ag_4(Py_3As)_4]^{5+}$ and $[Ag_2(PhPy_2As)_2(MeCN)_2]^{2+}$, using available X-ray coordinates. The single point computations have been carried out using the dispersion-corrected hybrid functional ωB97XD[5] incorporated in Gaussian-09 package.[1] The Douglas–Kroll–Hess 2nd order scalar relativistic calculations requested relativistic core Hamiltonian were carried out using the DZP-DKH basis sets[6–9] for all atoms. The topological analysis of the electron density distribution has been performed by using the Multiwfn program (version 3.7).[10]

§7. Temperature dependent excitation spectra of 1·CH₂Cl₂ and 2

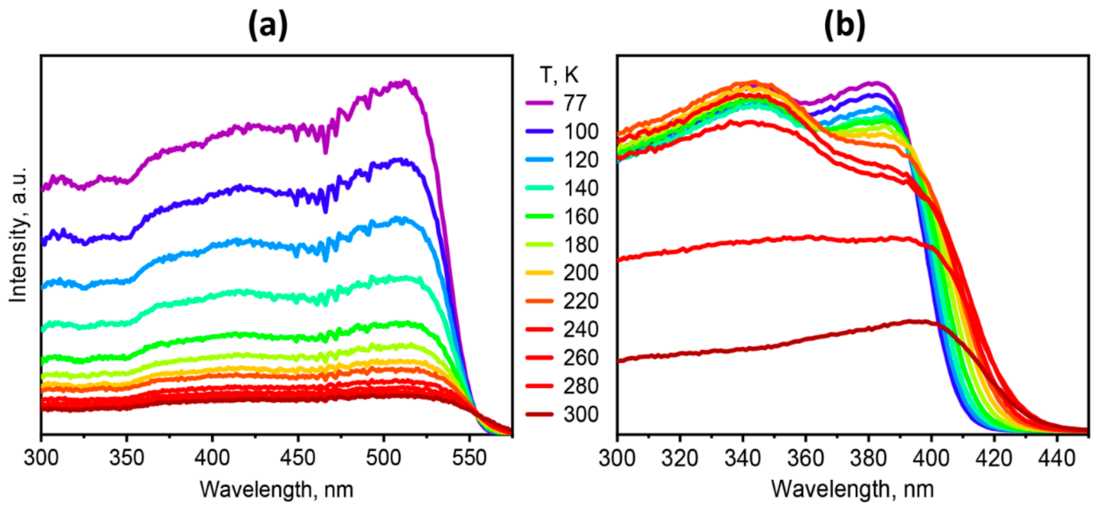

Figure S16. Temperature dependent excitation spectra of **1**·CH_2Cl_2 (a) and **2** (b) recorded at λ_{reg} = 595 and 520 nm, respectively.

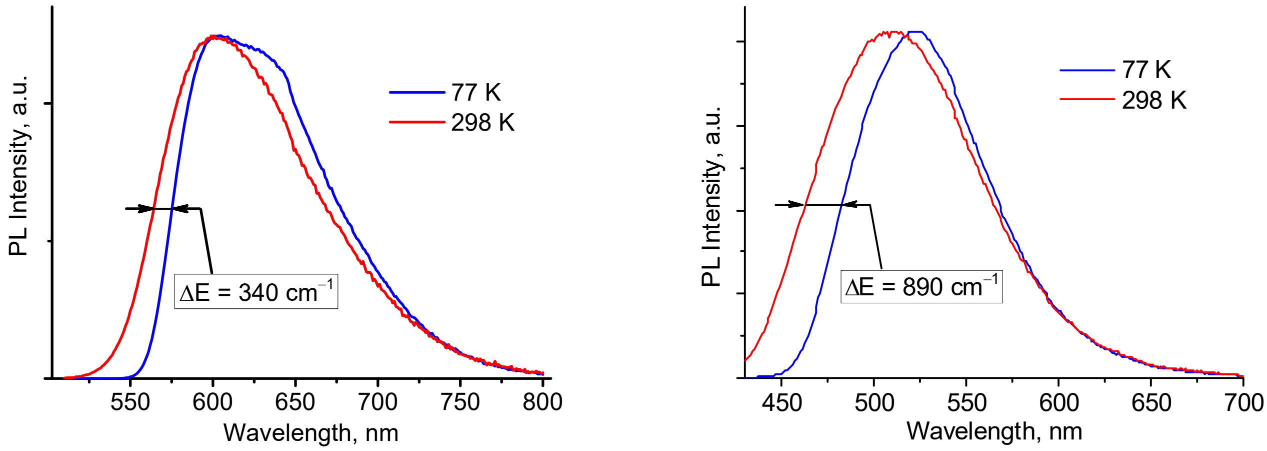

Figure S17. Red-shifting emission profile of **1**·CH_2Cl_2 (left) and **2** (right) upon cooling from 298 to 77 K.

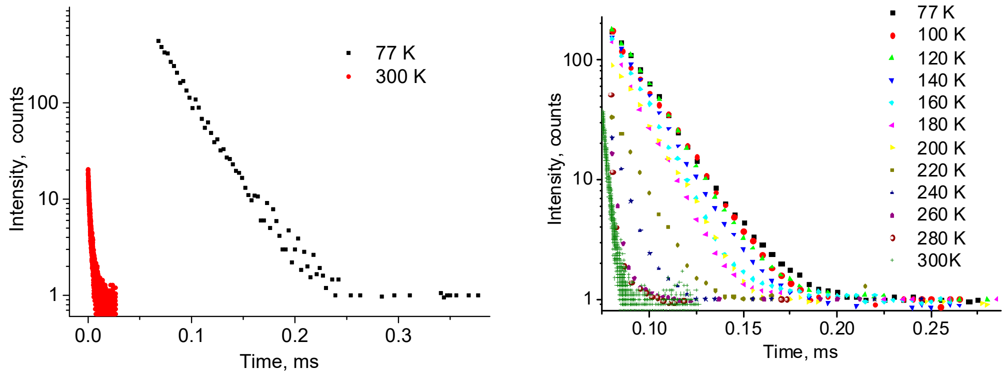

Figure S18. PL decay kinetics for **1**·CH_2Cl_2 (*left*) and **2** (*right*).

§8. References

1 M. Frisch, G.W. Trucks, H.B. Schlegel, G.E. Scuseria, M.A. Robb, J.R. Cheeseman, G. Scalmani, V. Barone, B. Mennucci, Ga. Petersson, et al., Gaussian 09, Revision C.01, Gaussian, Inc., Wallingford, CT, 2010.

2 (a) C. Adamo, V. Barone, *J. Chem. Phys.*, 1999, ***110***, 6158–6170; (b) J.P. Perdew, K. Burke, M. Ernzerhof, *Phys. Rev. Lett.*, 1996, ***77***, 3865–3868.

3 B.P. Pritchard, D. Altarawy, B. Didier, T.D. Gibson, T.L. Windus, *J. Chem. Inf. Model.*, 2019, ***59***, 4814–4820.

4 (a) Bauernschmitt, R. Ahlrichs, *Chem. Phys. Lett.*, 1996, ***256***, 454–464; (b) C. Van Caillie, R.D. Amos, *Chem. Phys. Lett.*, 1999, ***308***, 249-255; (c) G. Scalmani, M.J. Frisch, B. Mennucci, J. Tomasi, R. Cammi, V. Barone, *J. Chem. Phys.*, 2006, ***124***, 094107.

5 J.-D. Chaia, M. Head-Gordon, *Phys. Chem. Chem. Phys.*, **2008**, *10*, 6615–6620.

6 C.L. Barros, P.J.P. de Oliveira, F.E. Jorge, A. Canal Neto, M. Campos, *Mol. Phys.*, **2010**, *108*, 1965–1972.

7 F.E. Jorge, A. Canal Neto, G.G. Camiletti, S.F. Machado, *J. Chem. Phys.*, **2009**, *130*, 064108.

8 A. Canal Neto, F.E. Jorge, *Chem. Phys. Lett.*, **2013**, *582*, 158–162.

9 R.C. de Berrêdo, F.E. Jorge, *J. Mol. Struct. – Theochem*, **2010**, *961*, 107–112.

10 T. Lu, F. Chen, *J. Comput. Chem.*, **2012**, *33*, 580–592.
